# The Stereochemical Course of the α-Hydroxyphosphonate–Phosphate Rearrangement

**DOI:** 10.1002/chem.201406661

**Published:** 2015-06-08

**Authors:** Katharina Pallitsch, Alexander Roller, Friedrich Hammerschmidt

**Affiliations:** [a] Institute of Organic Chemistry, University of ViennaWähringerstraße 38, 1090 Vienna (Austria); [b] Institute of Inorganic Chemistry, University of ViennaWähringerstraße 42, 1090 Vienna (Austria)

**Keywords:** phosphates, phosphites, phosphonates, reaction mechanisms, rearrangement

## Abstract

The phosphonate–phosphate rearrangement is an isomerisation of α-hydroxyphosphonates bearing electron-withdrawing substituents at the α-carbon atom. We studied the stereochemical course of this rearrangement with respect to phosphorus. A set of four diastereomeric α-hydroxyphosphonates was prepared by a Pudovik reaction from two diastereomeric cyclic phosphites. The hydroxyphosphonates were separated and rearranged with Et_3_N as base. In analogy to trichlorphon, which was the first reported compound undergoing this rearrangement. All four hydroxyphosphonates could be rearranged to 2,2-dichlorovinyl phosphates. Single-crystal X-ray structure analyses of the α-hydroxyphosphonates and the corresponding phosphates allowed us to show that the rearrangement proceeds with retention of configuration on the phosphorus atom.

## Introduction

**Scheme 1:**
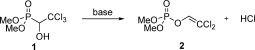
Base-catalysed rearrangement of trichlorphon to give DDVP.

Racemic dimethyl 2,2,2-trichloro-1-hydroxyethylphosphonate (**1**), also known as trichlorphon, is an organophosphonate that was introduced as an insecticide in the early 1950s.[1] Later it was used in medicine under the trademark “metrifonate” to treat schistosomiasis, a parasitic disease. This compound attracted the interest of the scientific community, when a unique kind of transformation was observed. Upon treatment with base, HCl was eliminated from the molecule (Scheme 1).[2]

The observed elimination product was *O*,*O*-dimethyl *O*-2,2-dichlorovinyl phosphate (**2**), also known as DDVP. This was the first reported α-hydroxyphosphonate–phosphate rearrangement.

**Scheme 2:**
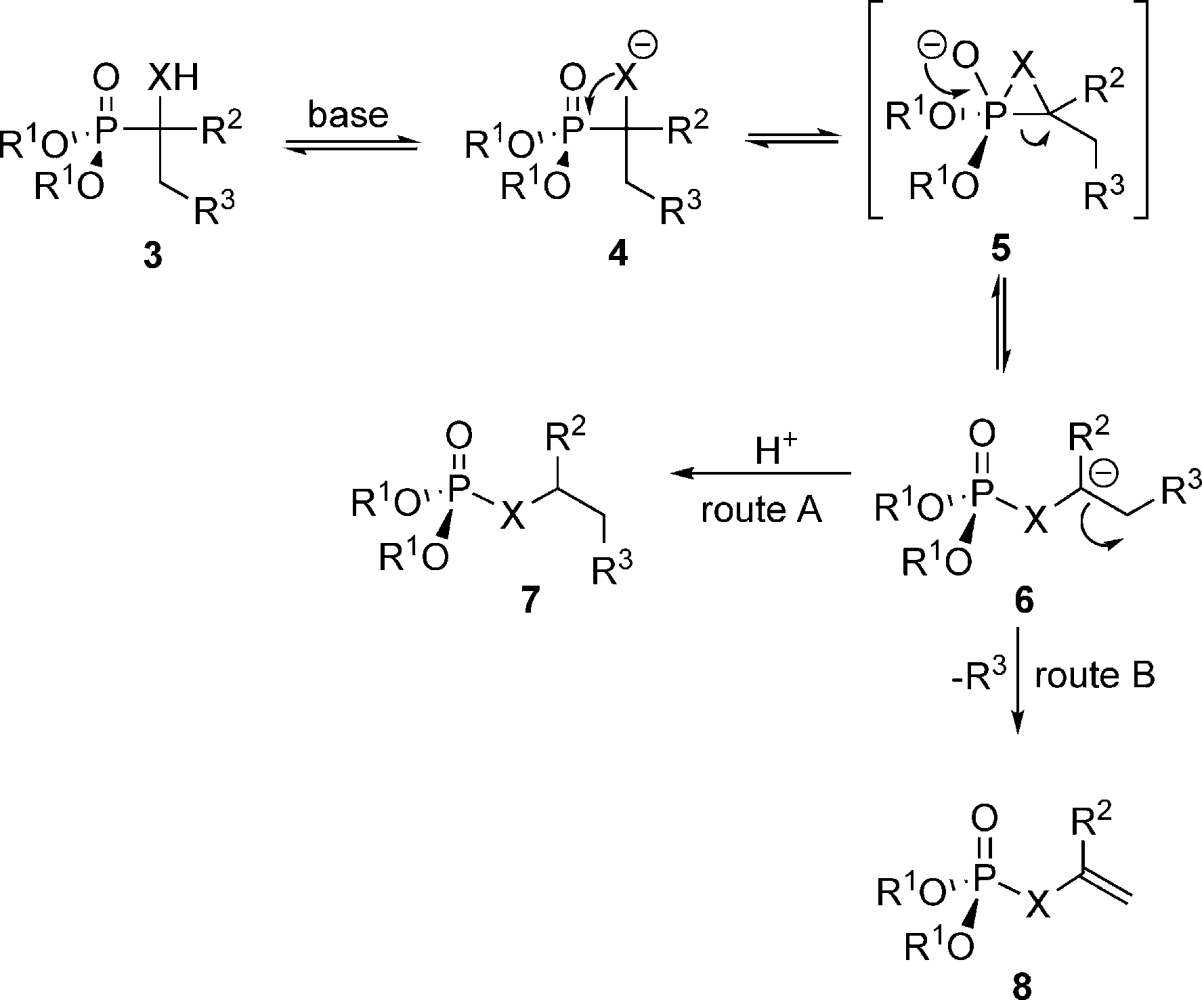
General reaction mechanism of the phosphonate–phosphate rearrangement. X=O, S or NH. Route A applies if R^3^ is not a leaving group and R^2^ is carbanion stabilising, route B applies if R^3^ is a leaving group.

Subsequently, other α-hydroxyphosphonates, not having a leaving group like chloride at the β-carbon atom, but an electron-withdrawing group on the α-carbon atom were shown to undergo the same transformation.[3,4] Therefore, a general reaction mechanism was proposed, which was later on proven by isotope labelling experiments (Scheme 2).[5]

After deprotonation of the α-hydroxyl group by the base, a nucleophilic attack of the oxyanion on the electrophilic phosphorus centre takes place. This induces P=C bond cleavage and either elimination of the leaving group R^3^ (if there is one at the C-2 atom, such as a halogen) or formation of a short-lived carbanion at the C-1 atom (if R^2^ is carbanion stabilising, such as a phenyl group), which is finally protonated.

The α-hydroxyphosphonate–phosphate rearrangement, as it is generally referred to, can be observed for α-hydroxyphosphonates bearing at least one electron-withdrawing substituent on the α-carbon atom next to phosphorus, such as aryl-, cyano- or halide residues.[6] Similarly, certain α-mercapto- and α-aminophosphonates undergo this isomerisation to give thiophosphates and phosphoramidates, respectively.

The corresponding retro-reaction, the phosphate–phosphonate rearrangement is also known. When a phosphate is treated with a stoichiometric amount of BuLi at low temperature, a proton is abstracted in α position to an oxygen atom. The resulting carbanion is configurationally stable and the ensuing P=C bond formation proceeds with retention of configuration at the involved carbon atom.[7]

This rearrangement is of industrial importance in the synthesis of DDVP (**2**). It was demonstrated that trichlorphon (**1**) has no physiological activity and that its effects are exclusively due to the non-enzymatic transformation to DDVP, which is the active agent. Therefore, trichlorphon was replaced by DDVP in some cases. However, trichlorphon can be used as a slow release formulation of DDVP,[1,8,9] which is an acetylcholinesterase inhibitor interfering with neuronal signal transmission.[3]

**Scheme 3:**
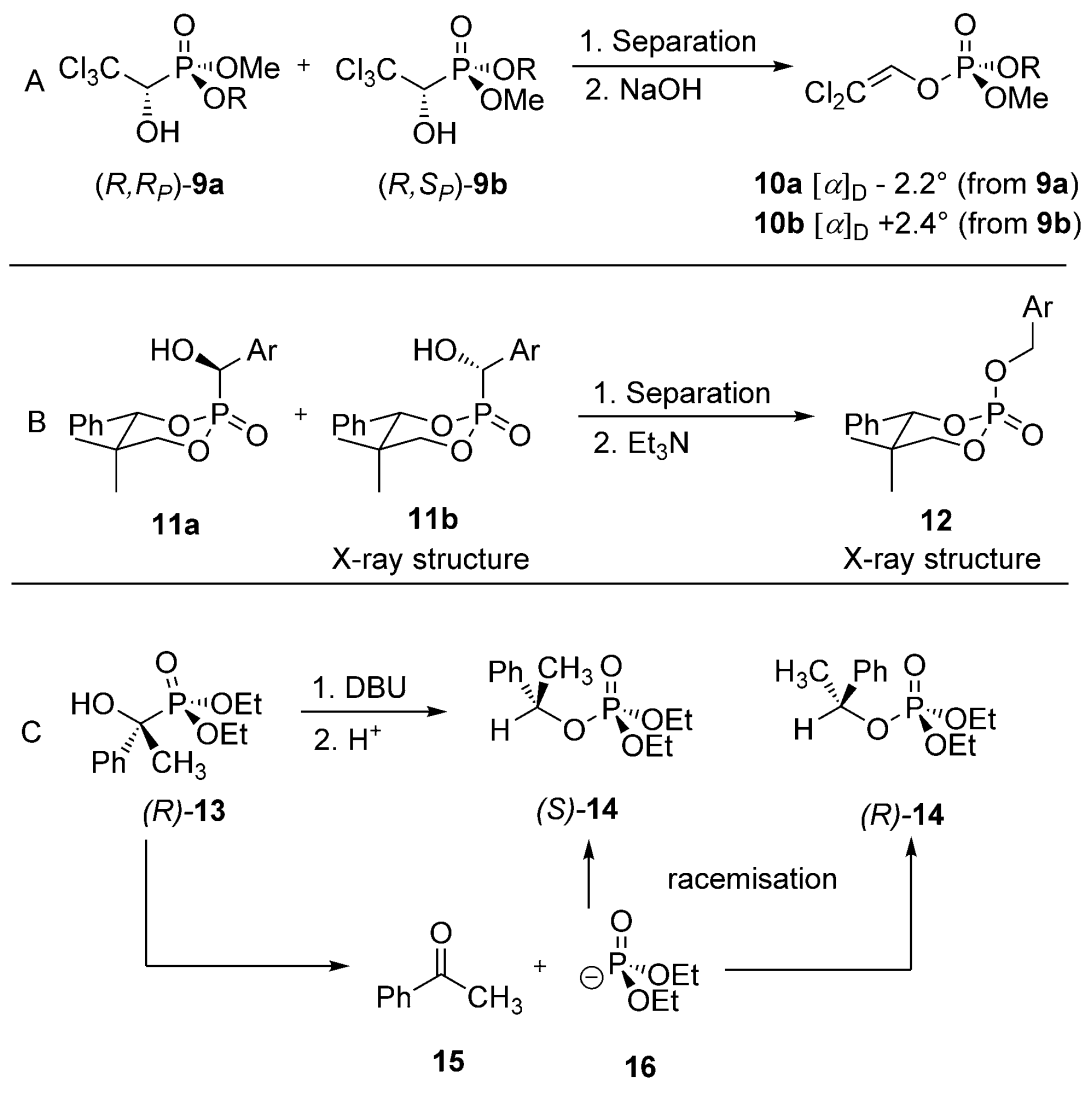
Previous work from A) Brienne et al.,[10] B) Jankowski et al.[11] and C) Hammerschmidt et al.[6] on the stereochemistry regarding the phosphorus and carbon atom.

If either the phosphorus centre or the neighbouring carbon atom involved in this reaction is bearing chiral information, the stereochemistry of the transformation can be studied. The α-hydroxyphosphonate–phosphate rearrangement was shown to proceed with retention of configuration with respect to the involved carbon centre by our group.[6] The reaction was carried out in a mixture of DMSO and water (95:5) by using 1,5-diazabicyclo[5.4.0]undec-5-ene (DBU) as a base (Scheme 3 C).

Because decomposition of the α-hydroxyphosphonates to the dialkyl phosphite anion **16** and ketone **15** is a fast—and thus troublesome—side reaction under those conditions, water is essential to quickly protonate the intermediately formed anion. The recombination of those two fragments to racemic α-hydroxyphosphonates is also base catalysed. This leads to the predominant formation of the racemic phosphate **14**. Still, the enantiomeric excess (*ee*) of the re-isolated starting material was high enough to deduce the stereochemical course of the reaction.

In a first attempt, Brienne and co-workers studied the stereochemistry at phosphorus by using derivatives of trichlorphon. They performed a chemical resolution of the racemic monomethyl ester of trichlorphon and esterified the enantiomerically pure isomers by using orthoformates to give the diastereomeric α-hydroxyphosphonates **9 a** and **9 b**. Separation by fractional crystallisation and subsequent single-crystal X-ray structure analysis allowed them to assign the absolute configuration of each diastereomer. Upon rearrangement by using NaOH as base, they obtained a pair of enantiomeric phosphates, that is, compounds **10 a** and **10 b** (each phosphonate giving only one phosphate), being chiral only due to phosphorus. Their experiments showed that the obtained phosphates were of opposite configuration as could be judged from the optical rotation. However, they could not crystallise them and thus could only show that the reaction is stereospecific, but whether it proceeds with retention or inversion of the configuration remained elusive at the time (Scheme 3 A).[10]

Recently, Jankowski and co-workers found that the rearrangement proceeds with retention of configuration on the involved phosphorus atom (Scheme 3 B).[11] However, the reaction was only performed with two diastereomeric α-hydroxyphosphonates **11 a** and **11 b**, bearing axial P=C bonds, derived from a six-membered cyclic phosphite. The two other α-hydroxyphosphonates, having equatorial P=C bonds, were not isomerised. Therefore, they could not give a general conclusion on the stereochemistry of the rearrangement.

## Results and Discussion

### General thoughts

We investigated whether the assumption made by Jankowski et al. that the α-hydroxyphosphonate–phosphate rearrangement proceeds with retention of the configuration at the phosphorus atom, is a general rule for all α-hydroxyphosphonates.

When the stereochemistry of the α-hydroxyphosphonate–phosphate rearrangement is studied, two main problems need to be overcome. Single-crystal X-ray structure analyses are necessary to assess the absolute configuration of the involved phosphorus atom before and after the rearrangement. The major difficulty in all prior attempts has been the poor tendency of phosphates to crystallise. Thus, first of all, it is essential to find a system of α-hydroxyphosphonates and phosphates giving crystalline compounds. Secondly, the decomposition of the α-hydroxyphosphonates to phosphites and aldehydes and the reverse process, both being base catalysed, have to be suppressed to prevent the formation of stereoisomers from homogeneous starting compounds.

In our current approach we envisaged the synthesis of four diastereomeric, cyclic α-hydroxyphosphonate analogues of trichlorphon. We wanted to make use of the HCl elimination during the rearrangement as a driving force for the reaction to prevent the decomposition of the α-hydroxyphosphonate to phosphite and aldehyde. Also, cyclic phosphates are known to crystallise more easily than acyclic ones. Using cyclic phophonates means that interconversion between the possible orientations of the P=C bonds in the molecules is impossible, as the rings fix their position. Thereby we wanted to overcome all prior problems.

Of our four diastereomeric hydroxyphosphonates two had axial and two had equatorial P=C bonds, in order to compare their behaviour during the rearrangement. Another key point of this current approach is the fact that upon rearrangement our substrates will give diastereomeric rather than enantiomeric phosphates. This will make their comparison and characterisation much easier compared to all former attempts.

### Synthetic strategy

We planned to synthesise all four stereoisomeric α-hydroxyphosphonates through a Pudovik reaction from an aldehyde and cyclic phosphites with an axial and an equatorial P=H bond, respectively. We already knew from other projects in our group that it is very easy to obtain six-membered cyclic phosphites by transesterification of the commercially available bis(2,2,2-trifluoroethyl) phosphite (**21**)[12] with 1,3-diols.[13] In case of a *C*_2_ symmetric, enantiomerically pure diol this leads to the clean formation of one cyclic phosphite. The phosphorus, in this case, is then a chirotopic, non-stereogenic centre.[14] Starting from an asymmetric 1,3-diol, a pair of diastereomeric phosphites is obtained. If those phosphites are reacted with a suitable aldehyde in Pudovik reactions, diastereomeric α-hydroxyphosphonates are obtained, which can be used to study the phosphonate–phosphate rearrangement.

**Scheme 4:**
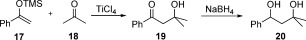
Synthesis of the racemic diol through a crossed aldol reaction followed by reduction.

Therefore, we chose 2-methyl-4-phenylbutane-2,4-diol (**20**) as our starting diol and improved its known synthesis.[15] It can be easily prepared by a TiCl_4_-mediated crossed aldol reaction of 1-phenyl-1-trimethylsiloxyethylene (**17**) with acetone (**18**) followed by reduction of the resulting crude β-hydroxy ketone **19** with sodium borohydride (Scheme 4).

**Scheme 5:**
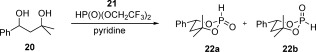
Synthesis of the two diastereomeric phosphites 22 a and 22 b through transesterification of compound 21.

The reaction is very reliable and furnished the diol in 75 % overall yield for both steps without purification of the intermediate ketone. The resulting diol **20** was used to transesterify bis(2,2,2-trifluoroethyl) phosphite (**21**) at room temperature in dry pyridine, which was the necessary base and the solvent at a time (Scheme 5).

This reaction proceeded quantitatively as judged by NMR spectroscopy and produced both diastereomeric cyclic phosphites **22 a** and **22 b** in a 1:1 ratio.

**Scheme 6:**
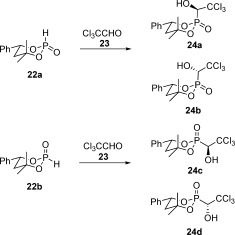
Synthesis of the diastereomeric α-hydroxyphosphonates 24 a–24 d for the rearrangement.

Initially, we wanted to continue our synthesis with this mixture of phosphites in order to keep the number of necessary purification steps as low as possible. Unfortunately, it was extremely difficult to separate all four diastereomeric α-hydroxyphosphonates **24 a**–**24 d** in the next step. Therefore, we separated the two phosphites and reacted them separately with anhydrous chloral (**23**) to give two α-hydroxyphosphonates each. As the two phosphites partially decomposed on silica gel, we lost some material and could only isolate a combined yield of 54 % of both diastereomers. Both were solids, but only the more polar diastereomer **22 b**[16] could be crystallised from hexanes/CH_2_Cl_2_ to give colourless needles, suitable for single-crystal X-ray structure analysis. It revealed an equatorial P=H bond for this diastereomer. However, we point out that the phosphorus centre keeps its tetrahedral configuration and distorts the six-membered ring. The chair conformation is rather flattened towards phosphorus. This makes it difficult to clearly define axial and equatorial positions. Therefore, we decided to have a look at the position of the phenyl substituent in the ring and use it as an internal reference point of equatorial position. The configuration of the other diastereomer, that is, compound **22 a**, which could not be determined by X-ray structure analysis, was underpinned by ^1^H NOESY NMR spectroscopy. The homogenous phosphites were then reacted separately with chloral at −30 °C catalysed by Et_3_N (Scheme 6).

Depending on the diastereomer, the starting phosphite was consumed to about 90 % after 5–7 h. In the case of the faster moving phosphite **22 a** the two α-hydroxyphosphonates were formed in a 1:1 ratio as judged by ^31^P NMR spectroscopy. They could be separated by flash chromatography. It is essential to remove Et_3_N quickly from the reaction mixture in order to avoid rearrangement of the product. Flash chromatography with a short column was thus performed first, followed by a second one with a longer column to separate the diastereomeric products. Both homogenous α-hydroxyphosphonates **24 a** and **24 b** are colourless solids. The faster moving diastereomer **24 a** could be crystallised from toluene/acetone at room temperature and furnished crystals suitable for X-ray structure analysis. It showed the P=C bond to be axially orientated, meaning that the P=H bond of the starting phosphite was replaced with retention of configuration. The other diastereomer derived from the same phosphite, that is, compound **24 b**, must also have an axial P=C bond, but be of opposite configuration at the newly formed stereogenic centre.

In the case of the more polar phosphite **22 b** the two α-hydroxyphosphonates were also formed in a 1:1 ratio (as could be judged from the final product yield). However, this ratio could not be determined from the crude reaction mixture in CH_2_Cl_2_. One of the two diastereomers, that is, compound **24 d**, has an extremely low solubility in all organic solvents except DMSO and DMF. Under the reaction conditions it crystallises. Therefore, NMR spectroscopy of the resulting turbid mixture is not representative for the actual diastereomeric ratio. However, this property made the separation of the two compounds very easy. They could be separated by digestion with ethyl acetate or dichloromethane. Again, both α-hydroxyphosphonates are crystalline solids. The less polar compound **24 c** yielded crystals suitable for X-ray structure determination, revealing that the equatorial P=H bond of the starting phosphite was replaced by an equatorial P=C bond. As each pair of hydroxyphosphonates was obtained from one phosphite they had to share the configuration of the phosphorus atom, as well as of the stereocentre in the six-membered ring. Consequently, each pair differs only in its configurations at the carbon atom bearing the hydroxyl group.

### The rearrangement

**Scheme 7:**
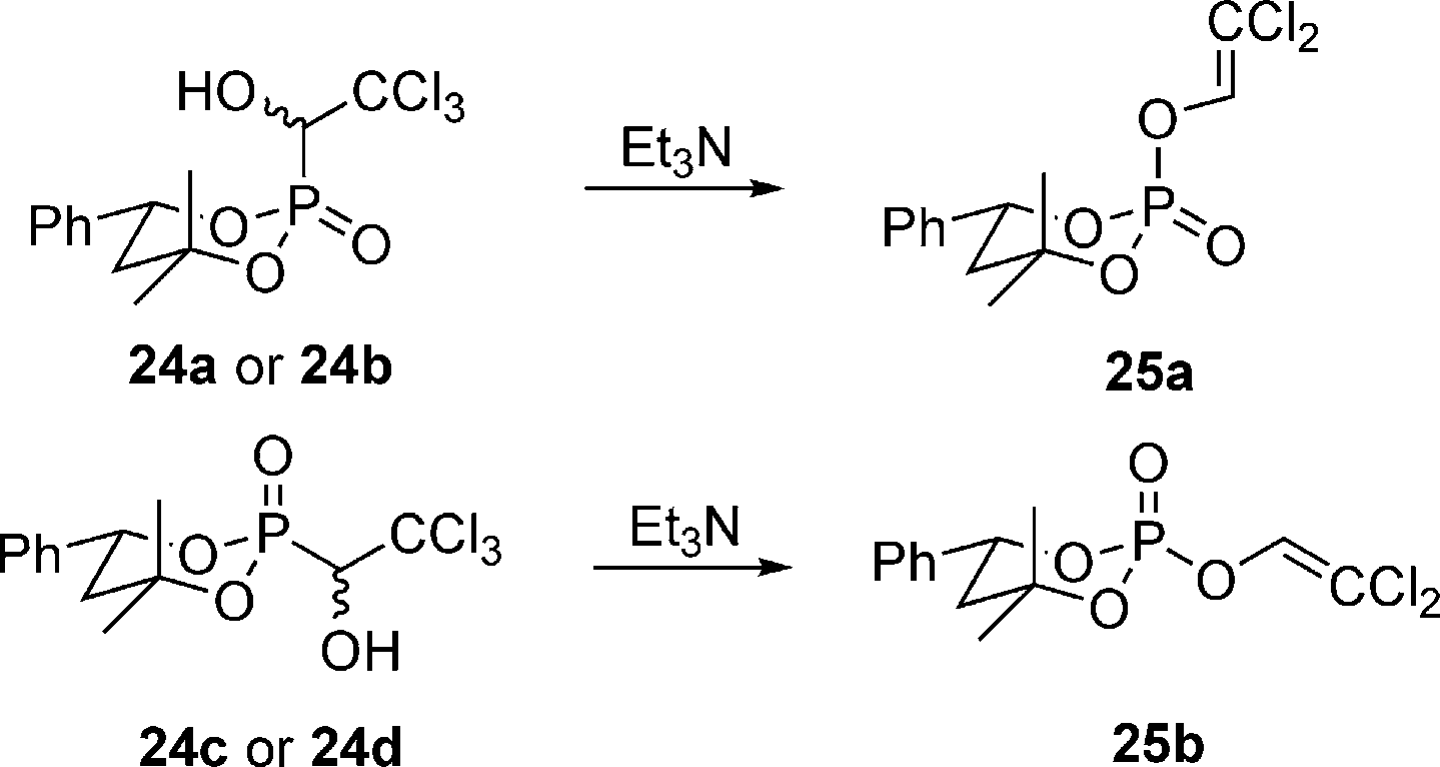
The rearrangement was performed in CHCl_3_ with Et_3_N at 0 °C.

Having all four desired α-hydroxyphosphonates in hand, as well as the corresponding crystal structures of two of them, namely compounds **24 a** and **24 c**, we rearranged them (Scheme 7).

We rearranged all four diastereomeric α-hydroxyphosphonates separately in CHCl_3_ at 0 °C by using Et_3_N as base in stoichiometric amounts. The reaction progress was monitored by ^31^P NMR spectroscopy. These experiments clearly demonstrated that the four phosphonates produce two different dichlorovinyl phosphates, compounds **25 a** and **25 b**. Furthermore, each pair of hydroxyphosphonates with the same configuration at the phosphorus atom gave exclusively one phosphate. This finding is an experimental proof for the stereospecific character of the reaction.

As anticipated, decomposition of the hydroxyphosphonates to chloral and the respective phosphite interfered with the rearrangement. Even though decomposition was observed to a high extent, the detected phosphite was always exclusively the one from which the studied hydroxyphosphonate was derived. Thus, no interconversion between the cyclic phosphites takes place by inversion of the configuration at the phosphorus atom. The amount of phosphite formed was dependent on the used base concentration. Surprisingly, no chloral could be detected by NMR spectroscopy. Further, no retro-reaction of phosphite and chloral could be observed at any time of the reaction, for which we did not find an explanation. We also tried to identify other possible side products. However, all our attempts to detect chloroform and carbon monoxide failed.[17]

NMR spectroscopic kinetics for the rearrangement of compound **24 a** can be found in the Supporting Information and are representative for all hydroxyphosphonates. Luckily, both vinylphosphates **25** turned out to be crystalline solids. A single-crystal X-ray structure analysis was performed of the less polar compound **25 a** having an axial dichlorovinyloxy substituent at the phosphorus atom. The isomeric cyclic phosphate **25 b** has therefore an equatorial dichlorovinyloxy substituent at the phosphorus atom.

Comparison of the crystal structures of the α-hydroxyphosphonates and the corresponding dichlorovinyl phosphates showed that the phosphorus atom does not change its configuration during the rearrangement. However, the six-membered ring is not a perfect chair due to the phosphorus atom as in the case of the phosphites. This leads to a distortion of the ring conformation of the α-hydroxyphosphonates, which is less dominant in case of the final phosphates. Still, the two molecules can be easily compared by studying the relative orientation of the phenyl and the 1-hydroxy-3,3,3-trichloroethyl or dichlorovinyloxy substituent on the phosphorus atom as already discussed. Thereby we were able to find that the α-hydroxyphosphonate–phosphate rearrangement proceeds with retention of configuration regarding the phosphorus atom in all four studied diastereomeric α-hydroxyphosphonates.

**Scheme 8:**
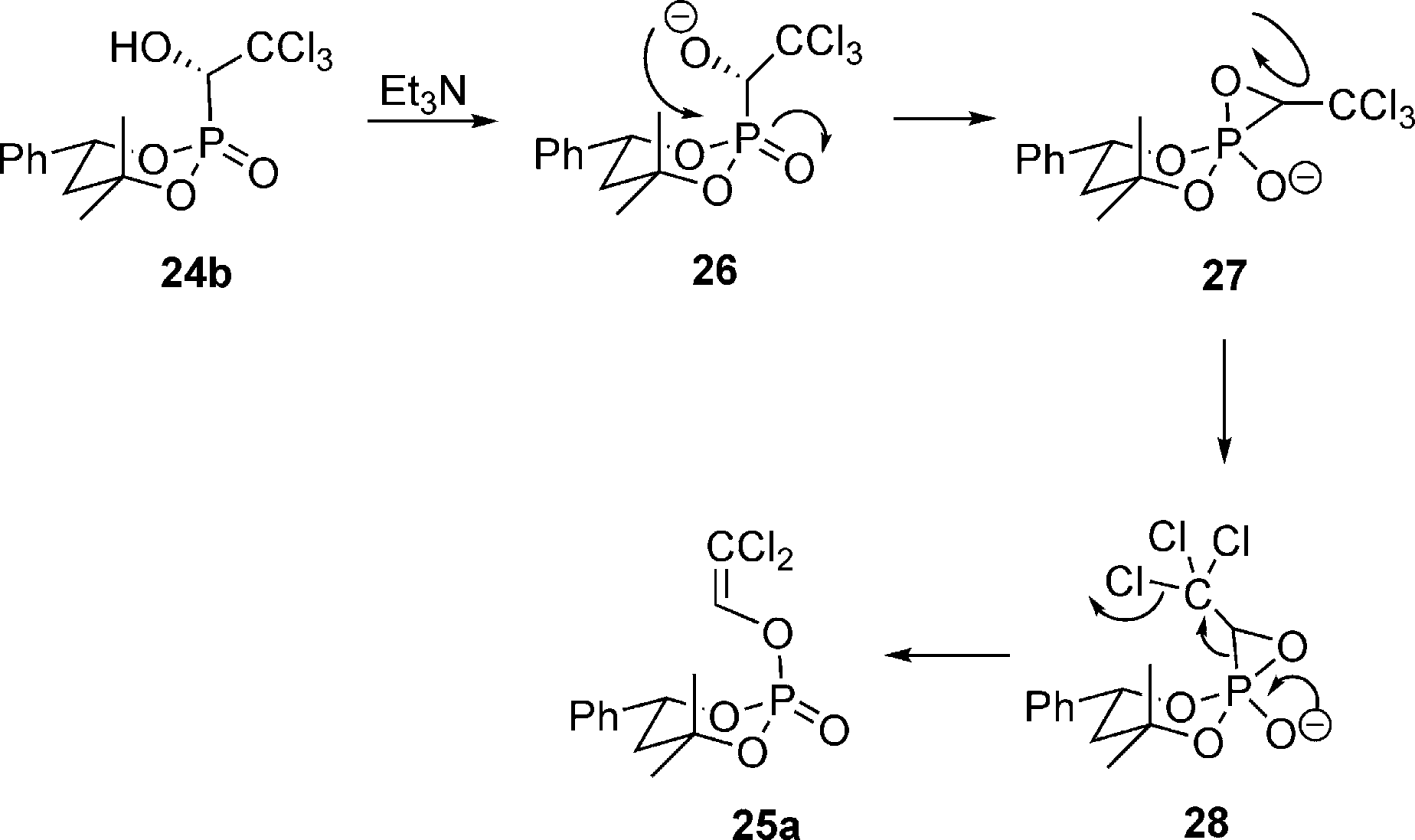
Proposed mechanism for the rearrangement of compound 24 b involving a turnstile rotation.

We assume that the reaction proceeds through a pentacoordinated phosphorus spirocentre, with both the P=C and the P=O bond still present in accordance to the mechanism described in Scheme 8. Upon treatment with base the α-hydroxyl group is deprotonated and attacks the phosphorus centre from the side opposing the P=O bond (intermediate **26**). The resulting spiro intermediate **27** cannot undergo a Berry pseudorotation as this is not possible for cyclic pentacoordinated phosphorus centres.[18] However, this intermediate can undergo a turnstile rotation leading to a configuration where the oxyanion and the P=C bond are opposing each other (intermediate **28**).[19] When the P=C bond breaks, the phosphorus atom gets again tetracoordinated and adopts its preferred tetrahedral structure. Thereby, the emerging phosphate **25 a** retains the same configuration as the starting α-hydroxyphosphonate.

Still, it has to be mentioned that our data, as well as those already published by Jankowski et al.[11] were obtained by using cyclic α-hydroxyphosphonates. Data for at least one acyclic α-hydroxyphosphonate are still missing.

### Further considerations

As there are some naturally occurring α-hydroxyphosphonic acids known, the retro-α-hydroxyphosphonate–phosphate rearrangement was once postulated as an alternative pathway to the rearrangement of phosphoenolpyruvate for the biosynthesis of phosphonates. It would have accounted for the P=C bond formation and the α-hydroxyl group introduction at a time.[20] This mechanism was ruled out by feeding experiments with *Tetrahymena thermophila* by using C-6 doubly deuterated glucose. However, recently a new pathway for the biodegradation of 2-aminoethylphosphonic acid (2-AEP) was discovered in marine microorganisms. Two enzymes are involved. The first one, PhnY, α-hydroxylates 2-AEP and the second one, PhnZ, effects the subsequent oxidative P=C bond cleavage.[21,22] It was suggested that an α-hydroxyphosphonate–phosphate-type rearrangement could form a mechanistic basis for the P=C bond cleavage.

## Conclusion

Diastereomeric, cyclic phosphites **22 a** and **22 b** were prepared, which further gave the four diastereomeric α-hydroxyphosphonate **24 a**–**24 d** analogues to trichlorphon. These could be easily rearranged to give the corresponding dichlorovinyl phosphates **25 a** and **25 b** by using Et_3_N as base. Four single-crystal X-ray structure analyses allowed the assignment of the configuration of the phosphorus atom in the diastereomeric phosphites, α-hydroxyphosphonates and phosphates. The combination of all these stereochemical datasets proves that the α-hydroxyphosphonate–phosphate rearrangement proceeds with retention of configuration regarding the involved phosphorus atom. The same is true for the α-hydroxyphosphonate formation from aldehyde and phosphite in a Pudovik reaction.

## Experimental Section

**General experimental**: ^1^H, ^13^C (in part *J* modulated) and ^31^P NMR spectra were recorded in CDCl_3_ on a Bruker Avance AV 400 (^1^H: 400.27, ^13^C: 100.65 and ^31^P: 162.03 MHz) or DRX 600 (^1^H: 600.13 MHz) spectrometer. Chemical shifts were referenced to residual CHCl_3_ (*δ*(H)=7.24 ppm), CDCl_3_ (*δ*(C)=77.23 ppm) and external H_3_PO_4_ (85 %). Chemical shifts (*δ*) are given in [ppm] and coupling constants (*J*) in [Hz]. IR spectra were run on a Bruker VERTEX 70IR spectrometer as ATR spectra. Melting points were determined on a Leica Galen III Reichert Thermovar instrument and are uncorrected.

TLC was carried out on 0.25 mm thick Merck plates, silica gel 60 F_254_. Spots were visualised by UV and/or dipping the plate into a solution of (NH_4_)_6_Mo_7_O_24_**⋅**4 H_2_O (25.0 g) and Ce(SO_4_)_2_**⋅**4 H_2_O (1.0 g) in 10 % aqueous H_2_SO_4_ solution (500 mL), followed by heating with a heat gun. Flash (column) chromatography was performed with Merck silica gel 60 (230–400 mesh).

Pyridine was dried by heating to reflux over powdered CaH_2_, then distilled and stored over molecular sieves (4 Å). Dichloromethane was dried by passing through aluminum oxide 90 active neutral (0.063–0.200 mm, activity I) and stored over molecular sieves (3 Å). Bis(2,2,2-trifluoroethyl) phosphite was distilled under reduced pressure (b.p. 48–50 °C/9 mm; lit.:[12] 43–44 °C/2 mm). All other chemicals were used as purchased from Sigma–Aldrich, Acros, Fluka or Merck.

**(±)-1-Methyl-4-phenylbutane-2,4-diol (20)**: Acetone (0.639 g, 0.87 mL, 11.0 mmol) was added to a solution of TiCl_4_ (2.087 g, 1.21 mL, 11.0 mmol) in dry CH_2_Cl_2_ (60 mL) at 0 °C. Silylenol ether **17** (1.926 g, 2.03 mL, 10.0 mmol) was then added dropwise and stirring was continued at 0 °C. After 1.5 h the brownish red solution was poured into ice-cold water (200 mL). The biphasic mixture was stirred for 15 min whereupon it turned pale yellow. The phases were separated and the aqueous layer was extracted with CH_2_Cl_2_ (3×30 mL). The combined organic layers were subsequently washed with saturated aqueous NaHCO_3_ solution (2×60 mL) and saturated aqueous NaCl solution (1×40 mL) and then dried (MgSO_4_). The solution was concentrated in vacuo. The residue was dissolved in dry ethanol (20 mL) and added dropwise to a stirred solution of NaBH_4_ (0.946 g, 25.0 mmol) in dry ethanol (20 mL). The reaction mixture was stirred at room temperature for 19 h. Water (20 mL), pentaerythritol (4.085 g, 30.0 mmol) and NaOH (0.600 g, 15.0 mmol) were added whereupon a white precipitate formed. Vigorous stirring was continued for 3 h. Then, the reaction mixture was extracted with EtOAc (4×20 mL). The combined organic layers were washed with water (3×20 mL), dried (MgSO_4_) and concentrated under reduced pressure. The crude product was purified by flash chromatography (hexanes/EtOAc 3:1, *R*_f_ (diol)=0.20) to give the desired diol (±)-**20** (1.25 g, 6.9 mmol, 69 %) as a colourless oil. Crystallisation from hexanes furnished colourless crystals. M.p. 65–67 °C. The spectra were in accordance with those reported in the literature.[23]

(2S*,6R*)-4,4-Dimethyl-6-phenyl-1,3,2-dioxaphosphinane-2-oxide (22 a) and (2R*,6R*)-4,4-dimethyl-6-phenyl-1,3,2-dioxaphosphinane-2-oxide (22 b): The racemic diol (±)-**20** (1.420 g, 7.9 mmol) was dissolved in dry pyridine (20 mL) under argon at room temperature. Bis(2,2,2-trifluoroethyl) phosphite (2.332 g, 1.51 mL, 9.5 mmol) was added and stirring was continued for 2.5 h. Pyridine was removed by azeotropic drying with toluene. ^1^H NMR spectroscopy of the crude reaction mixture showed a 1:1 ratio of the resulting diastereomeric phosphites. The crude product was flash chromatographed (CH_2_Cl_2_/acetone 20:1; *R*_f_ (**22 a**)=0.43, *R*_f_ (**22 b**)=0.38) and yielded the less polar phosphite **22 a** (0.572 g, 2.5 mmol, 32 %) and the more polar phosphite **22 b** (0.391 g, 1.7 mmol, 22 %) as colourless solids.

Crystallisation of compound **22 b** from hexanes/CH_2_Cl_2_ at room temperature gave colourless needles. Phosphite **22 a** could not be re-crystallised.

*Compound **22 a***: M.p. 52–55 °C; ^1^H NMR (400.27 MHz, CDCl_3_, 25 °C): *δ*=7.43–7.30 (m, 5 H; CH_arom_), 7.14 (d, ^1^*J*(H,P)=682.8 Hz, 1 H; H-P), 5.48 (td, *J*=2.3, 12.0 Hz, 1 H; OCH), 2.11 (ABXP system, A-part: dd, ^2^*J*(H,H)=14.8, ^3^*J*(H,H)*=*12.0 Hz, B-part: td, ^2^*J*(H,H)=14.8, ^3^*J*(H,H)=^4^*J*(H,P)*=*2.3 Hz, 2 H; CH_2_), 1.64 (s, 3 H; CH_3_), 1.50 ppm (d, ^4^*J*(H,P)=1.8 Hz, 3 H; CH_3_); ^13^C NMR (100.66 MHz, CDCl_3_, 25 °C, *J* mod): *δ*=138.9 (d, ^3^*J*(C,P)=8.6 Hz, C_arom_), 129.02 (s, CH_arom_), 128.96 (s, CH_arom_), 125.8 (s, CH_arom_), 83.2 (d, ^2^*J*(C,P)=6.9 Hz, C-O), 76.4 (d, ^2^*J*(C,P)=5.0 Hz, CH-O), 45.9 (d, ^3^*J*(C,P)=6.7 Hz, CH_2_), 31.9 (d, ^3^*J*(C,P)=5.3 Hz, CH_3_), 26.7 ppm (d, ^3^*J*(C,P)=1.6 Hz, CH_3_); ^31^P NMR (162.03 MHz, CDCl_3_, 25 °C): *δ*=−2.71 ppm (s); IR (ATR): $\tilde \nu $
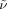
=2980, 2924, 2407, 1376, 1283, 1210, 1186, 1138, 1053, 1024, 972, 948 cm^−1^; HRMS (ESI-QTOF): calcd 249.0651 [*M*+Na]^+^; found: 249.0662; calcd: 475.1410 [*M*_2_+Na]^+^; found: 475.1418; elemental analysis calcd (%) for C_11_H_15_O_3_P: C 58.41, H 6.68; found: C 58.17, H 6.90.

*Compound **22 b***: M.p. 73–78 °C (ground crystals); 83–89 °C (single needle); ^1^H NMR (400.27 MHz, CDCl_3_, 25 °C): *δ*=7.43–7.30 (m, 5 H; CH_arom_), 7.15 (d, ^1^*J*(H,P)=717.7 Hz, 1 H; H-P), 5.68 (dt, *J*=12.1, 2.6 Hz, 1 H; OCH), 2.11 (ABXP system, A-part: dd, ^2^*J*(H,H)=14.8, ^3^*J*(H,H)*=*12.1 Hz, B-part: td, ^2^*J*(H,H)=14.8, ^3^*J*(H,H)=2.6, ^4^*J*(H,H)*=*1.2 Hz, 2 H; CH_2_), 1.72 (s, 3 H; CH_3_), 1.48 ppm (d, ^4^*J*(H,H)*=*1.2 Hz, 3 H; CH_3_); ^13^C NMR (100.66 MHz, CDCl_3_, 25 °C, *J* mod): *δ*=139.3 (d, ^3^*J*(C,P)=7.8 Hz, C_arom_), 129.0 (s, CH_arom_), 128.9 (s, CH_arom_), 125.7 (s, CH_arom_), 82.2 (d, ^2^*J*(C,P)=7.4 Hz, C-O), 75.9 (d, ^2^*J*(C,P)=6.4 Hz, CH-O), 45.5 (d, ^3^*J*(C,P)=7.9 Hz, CH_2_), 31.4 (d, ^3^*J*(C,P)=6.0 Hz, CH_3_), 28.0 ppm (s, CH_3_); ^31^P NMR (162.03 MHz, CDCl_3_, 25 °C): *δ*=−1.52 ppm (s); IR (ATR): $\tilde \nu $
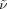
=3491, 2981, 2421, 1266, 1141, 1063, 1027, 971, 940 cm^−1^; HRMS (ESI-QTOF): calcd: 249.0651 [*M*+Na]^+^; found: 249.0656; calcd: 475.1410 [*M*_2_+Na]^+^; found: 475.1423; elemental analysis calcd (%) for C_11_H_15_O_3_P: C 58.41, H 6.68; found: C 58.51, H 6.59.

(2S*,6R*,1′R*)-4,4-Dimethyl-6-phenyl-2-(2,2,2-trichloro-1-hydroxyethyl)-1,3,2-dioxaphosphinane-2-oxide (24 a) and (2S*,6R*,1′S*)-4,4-dimethyl-6-phenyl-2-(2,2,2-trichloro-1-hydroxyethyl)-1,3,2-dioxaphosphinane-2-oxide (24 b): Phosphite **22 a** (0.519 g, 2.3 mmol) was dissolved in dry CH_2_Cl_2_ (7.5 mL) under argon at room temperature and the reaction mixture was cooled to −30 °C. Anhydrous chloral (0.508 g, 0.33 mL, 3.45 mmol) and Et_3_N (0.116 g, 0.16 mL, 1.15 mmol) were added and stirring was continued at −30 °C. After 7 h the reaction mixture was directly applied to a short flash chromatography column (CH_2_Cl_2_/acetone 14:1) to remove Et_3_N. Separation of the two diastereomeric α-hydroxyphosphonates was achieved by flash chromatography (CH_2_Cl_2_/acetone 20:1, *R*_f_ (**24 a**)=0.26, *R*_f_ (**24 b**)=0.19). The α-hydroxyphosphonates **24 a** (0.271 g, 0.73 mmol, 32 %) and **24 b** (0.245 g, 0.67 mmol, 29 %) were obtained as colourless, crystalline solids. α-Hydroxyphosphonate **24 a** could be crystallised from toluene/acetone at room temperature to yield colourless needles suitable for single-crystal X-ray structure analysis.

*Compound **24 a***: M.p. 162–163 °C (ground crystals, decomp. directly after melting), 173–175 °C (single needle); ^1^H NMR (400.27 MHz, CDCl_3_, 25 °C): *δ*=7.41–7.30 (m, 5 H; CH_arom_), 5.66 (td, ^3^*J*(H,H)=^3^*J*(H,P)=11.8, ^3^*J*(H,H)=2.3 Hz, 1 H; CH-O), 4.49 (dd, ^3^*J*(H,H)=8, ^3^*J*(H,P)=11.8 Hz, 1 H; OH), 4.01 (dd, ^3^*J*(H,H)=8.0, ^2^*J*(H,P)=16.8 Hz, 1 H; CH-P), 2.28 (ABXP system, A-part: dd, ^2^*J*(H,H)=15.0, ^3^*J*(H,H)*=*11.8 Hz, B-part: td, ^2^*J*(H,H)=15.0, ^3^*J*(H,H)=^4^*J*(H,H)=2.3 Hz, 2 H; CH_2_), 1.66 (s, 3 H; CH_3_), 1.57 ppm (s, 3 H; CH_3_); ^13^C NMR (100.61 MHz, CDCl_3_, 25 °C, not *J* mod): *δ*=138.9 (d, ^3^*J*(C,P)=7.1 Hz, C_arom_), 129.04 (s, CH_arom_), 128.98 (s, CH_arom_), 125.9 (s, CH_arom_), 98.1 (d, ^2^*J*(C,P)=8.8 Hz, CCl_3_), 84.6 (d, ^2^*J*(C,P)=8.5 Hz, C-O), 81.0 (d, ^1^*J*(C,P)=159.6 Hz, CH-P), 78.4 (d, ^2^*J*(C,P)=6.7 Hz, O-CH), 46.7 (d, ^3^*J*(C,P)=9.1 Hz, CH_2_), 31.9 (d, ^3^*J*(C,P)=2.8 Hz, CH_3_), 27.9 ppm (d, ^3^*J*(C,P)=3.6 Hz, CH_3_); ^31^P NMR (162.03 MHz, CDCl_3_; 25 °C): *δ*=8.10 ppm (s); IR (ATR): $\tilde \nu $
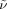
=3152, 2982, 1263, 1209, 1136, 1100, 1053, 1026, 987, 968 cm^−1^; HRMS (ESI-QTOF): calcd: 371.9846; found: 371.9841; elemental analysis calcd (%) for C_13_H_16_Cl_3_O_4_P: C 41.79, H 4.32; found: C 41.74, H 4.34.

*Compound **24 b***: M.p. 171–173 °C; ^1^H NMR (400.27 MHz, CDCl_3_, 25 °C): *δ*=7.47–7.35 (m, 5 H; CH_arom_), 5.69 (td, ^3^*J*(H,H)=^3^*J*(H,P)=11.8, ^3^*J*(H,H)=2.4 Hz, 1 H; CH-O), 4.47 (d, ^2^*J*(H,P)=12.1 Hz, 1 H; CH-P), 2.35 (ABXP system, A-part: dd, ^2^*J*(H,H)=15.0, ^3^*J*(H,H)*=*11.8 Hz, B-part: td, ^2^*J*(H,H)=15.0, *J*=2.4 Hz, 2 H; CH_2_), 1.70 (s, 3 H; CH_3_), 1.63 ppm (s, 3 H; CH_3_); ^13^C NMR (100.61 MHz, CDCl_3_, 25 °C, *J* mod): *δ*=139.1 (d, ^3^*J*(C,P)=6.9 Hz, C_arom_), 129.02 (s, CH_arom_), 128.95 (s, CH_arom_), 125.9 (s, CH_arom_), 84.6 (d, ^2^*J*(C,P)=8.6 Hz, C-O), 81.1 (d, ^2^*J*(C,P)=162.7 Hz, CH-P), 78.4 (d, ^2^*J*(C,P)=6.8 Hz, O-CH), 46.8 (d, ^3^*J*(C,P)=9.5 Hz, CH_2_), 32.0 (d, ^3^*J*(C,P)=2.4 Hz, CH_3_), 28.0 ppm (d, ^3^*J*(C,P)=3.8 Hz, CH_3_); (CCl_3_ not detected); ^31^P NMR (162.03 MHz, CDCl_3_, 25 °C): *δ*=7.53 ppm (s); IR (ATR): $\tilde \nu $
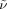
=3393, 3160, 2986, 1577, 1456, 1390, 1343, 1241, 1223, 1134, 1097, 1053, 988 cm^−1^; HRMS (ESI-QTOF): calcd: 371.9846; found: 371.9836.

(2R*,6R*,1′S*)-4,4-Dimethyl-6-phenyl-2-(2,2,2-trichloro-1-hydroxyethyl)-1,3,2-dioxaphosphinane-2-oxide (24 c) and (2R*,6R*,1′R*)-4,4-dimethyl-6-phenyl-2-(2,2,2-trichloro-1-hydroxyethyl)-1,3,2-dioxaphosphinane-2-oxide (24 d): Phosphite **22 b** (0.331 g, 1.5 mmol) was reacted with chloral as described above for phosphite **22 a**. The reaction mixture became turbid during the reaction time. After 7 h the crystals consisting mainly of diastereomer **24 d** were collected by filtration and the filtrate was directly applied to a column for chromatography (CH_2_Cl_2_/acetone 14:1) to remove Et_3_N. As there were large differences in the solubility these two α-hydroxyphosphonates could be separated by treating them with a 1:1 mixture of hexanes/CH_2_Cl_2_ in the ultrasonic bath for 20 min. Compound **24 c** dissolved easily in this mixture, whereas compound **24 d** remained as a solid residue (TLC in CH_2_Cl_2_/acetone 14:1, *R*_f_ (**24 c**)=0.21, *R*_f_ (**24 d**)=0.13). The residue was combined with the solid obtained by filtration of the reaction mixture. The two diastereomers **24 c** (0.152 g, 0.41 mmol, 27 %) and **24 d** (0.169 g, 0.45 mmol, 30 %) were obtained as colourless solids. Crystallisation of compound **24 c** from CH_2_Cl_2_/acetone at room temperature yielded crystals suitable for single-crystal structure X-ray analysis.

*Compound **24 c***: M.p. 156–158 °C (decomp. directly after melting); ^1^H NMR (400.27 MHz, CDCl_3_, 25 °C): *δ*=7.42–7.31 (m, 5 H; CH_arom_), 5.74 (td, ^3^*J*(H,H)=^3^*J*(H,P)=12.3, ^3^*J*(H,H)=2.2 Hz, 1 H; CH-O), 4.51 (dd, ^3^*J*(H,H)=6.8, ^3^*J*(H,P)=12.1 Hz, 1 H; either OH or CH-P, 3.90 (dd, ^3^*J*(H,H)=6.8, ^2^*J*(H,P)=16.3 Hz, 1 H; either CH-P or OH), 2.15 (ABXP system, A-part: dd, ^2^*J*(H,H)=14.7, ^3^*J*(H,H)*=*12.3 Hz, B-part: td, ^2^*J*(H,H)=14.7, ^3^*J*(H,H)=^4^*J*(H,H)=2.2 Hz, 2 H; CH_2_), 1.76 (s, 3 H; CH_3_), 1.51 ppm (d, *J=*2.1 Hz, 3 H; CH_3_); ^13^C NMR (100.61 MHz, CDCl_3_, 25 °C): *δ*=138.9 (d, ^3^*J*(C,P)=8.7 Hz, C_arom_), 129.1 (s, CH_arom_), 129.0 (s, CH_arom_), 125.9 (s, CH_arom_), 98.1 (d, ^2^*J*(C,P)=8.5 Hz, CCl_3_), 84.1 (d, ^2^*J*(C,P)=8.8 Hz, C-O), 80.3 (d, ^1^*J*(C,P)=163.8 Hz, CH-P), 76.9 (d, ^2^*J*(C,P)=6.6 Hz, O-CH), 44.5 (d, ^3^*J*(C,P)=5.4 Hz, CH_2_), 31.0 (d, ^3^*J*(C,P)=6.9 Hz, CH_3_), 27.8 ppm (s, CH_3_); ^31^P NMR (162.03 MHz, CDCl_3_, 25 °C): *δ*=8.68 ppm (s); IR (ATR): $\tilde \nu $
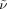
=3183, 2980, 2919, 2850, 1376, 1254, 1208, 1138, 1101, 1035, 970 cm^−1^; HRMS (ESI-QTOF): calcd: 372.99246 [*M*+H]^+^; found: 372.9932; calcd: 394.97440 [*M*+Na]^+^; found: 394.9753; elemental analysis calcd (%) for C_13_H_16_Cl_3_O_4_P: C 41.79, H 4.32; found: C 41.89, H 4.37.

*Compound **24 d***: M.p. 167–170 °C (decomp. after melting); ^1^H NMR (400.27 MHz, CDCl_3_, 25 °C): *δ*=7.41–7.32 (m, 5 H; CH_arom_), 5.74 (d, ^3^*J*(H,H)=12.1 Hz, 1 H; CH-O), 4.53 (d, ^3^*J*(H,H)=12.1 Hz, 1 H; OH), 3.64 (br s, 1 H; CH-P), 2.17 (ABXP system, A-part: dd, ^2^*J*(H,H)=14.7, ^3^*J*(H,H)*=*12.1 Hz, B-part: td, ^2^*J*(H,H)=14.7, ^3^*J*(H,H)=^4^*J*(H,H)=2.2 Hz, 2 H; CH_2_), 1.76 (s, 3 H; CH_3_), 1.52 ppm (d, *J=*1.9 Hz, 3 H; CH_3_); ^13^C NMR (100.61 MHz, CDCl_3_, 25 °C, *J* mod): *δ*=138.9 (d, ^3^*J*(C,P)=8.3 Hz, C_arom_), 129.1 (s, CH_arom_), 129.0 (s, CH_arom_), 126.0 (s, CH_arom_), 83.9 (d, ^2^*J*(C,P)=8.3 Hz, C-O), 80.2 (d, ^1^*J*(C,P)=162.4 Hz, CH-P), 76.9 (d, ^2^*J*(C,P)=6.9 Hz, O-CH), 44.5 (d, ^3^*J*(C,P)=5.1 Hz, CH_2_), 31.2 (d, ^3^*J*(C,P)=7.1 Hz, CH_3_), 27.8 ppm (s, CH_3_); (CCl_3_ not detected); ^31^P NMR (162.03 MHz, CDCl_3_, 25 °C): *δ*=8.37 ppm (s); IR (ATR): $\tilde \nu $
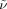
=3117, 2978, 2934, 1210, 1140, 1099, 1062, 1038, 987, 967 cm^−1^; HRMS (ESI-QTOF): calcd: 372.99246 [*M*+H]^+^; found: 372.9932; calcd: 394.97440 [*M*+Na]^+^; found: 394.9753; elemental analysis calcd (%) for C_13_H_16_Cl_3_O_4_P: C 41.79, H 4.32; found: C 41.58, H 4.26.

(2S*,6R*)-2-(2,2-Dichlorovinyloxy)-4,4-dimethyl-6-phenyl-1,3,2-dioxaphosphinane-2-oxide (25 a) and (2R*,6R*)-2-(2,2-dichlorovinyloxy)-4,4-dimethyl-6-phenyl-1,3,2-dioxaphosphinane-2-oxide (25 b): α-Hydroxyphosphonate **24 a** or **24 b** (0.041 g, 0.11 mmol) was dissolved in CDCl_3_ (2.5 mL) at 0 °C and Et_3_N (0.013 g, 0.02 mL, 0.13 mmol) was added. The solution was allowed to come to room temperature. The reaction progress was monitored by ^31^P/^1^H NMR spectroscopy. After 23 h at room temperature the rearrangement was completed to 40–45 mol % as judged by NMR spectroscopy.[24] The reaction mixture was concentrated in vacuo and applied to a column for flash chromatography (hexanes/EtOAc 2:1, *R*_f_ (**25 a**)=0.76) to obtain phosphate **25 a** as a colourless solid (0.011 g, 0.03 mmol, 30 %). Crystallisation from diisopropyl ether gave thin needles, suitable for single-crystal X-ray structure analysis.

Phosphate **25 b** was obtained by the same procedure starting from α-hydroxyphosphonate **24 c** or **24 d** as a crystalline solid. Flash chromatography was used for purification (hexanes/EtOAc 2:1, *R*_f_ (**25 b**)=0.41).

*Compound **25 a***: M.p. 79–81 °C; ^1^H NMR (400.27 MHz, CDCl_3_, 25 °C): *δ*=7.43–7.32 (m, 5 H; CH_arom_), 7.10 (d, ^3^*J*(H,P)=6.1 Hz, 1 H; =CH=), 5.60 (d, ^3^*J*(H,H)=12.0 Hz, 1 H; OCH), 2.10 (ABXP system, A-part: dd, ^2^*J*(H,H)=14.9, ^3^*J*(H,H)*=*12.0 Hz, B-part: td, ^2^*J*(H,H)=14.9, ^3^*J*(H,H)*=*2.3 Hz, 2 H; CH_2_), 1.71 (s, 3 H; CH_3_), 1.51 ppm (d, *J*=2.8 Hz, 3 H, CH_3_); ^13^C NMR (100.62 MHz, CDCl_3_, 25 °C, not *J* mod): *δ*=138.4 (d, ^3^*J*(C,P)=10.2 Hz, C_arom_), 134.3 (d, ^2^*J*(C,P)=3.9 Hz, =HC=), 129.2 (s, CH_arom_), 129.1 (s, CH_arom_), 125.8 (s, CH_arom_), 113.2 (d, ^3^*J*(C,P)=14.6 Hz,=CCl_2_), 85.8 (d, ^2^*J*(C,P)=8.1 Hz, C-O), 78.5 (d, ^2^*J*(C,P)=6.1 Hz, O-CH), 45.4 (d, ^3^*J*(C,P)=5.1 Hz, CH_2_), 31.6 (d, ^3^*J*(C,P)=9.5 Hz, CH_3_), 27.9 ppm (s, CH_3_); ^31^P NMR (162.03 MHz, CDCl_3_, 25 °C): *δ*=−13.01 ppm (s); IR (ATR): $\tilde \nu $
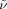
=3069, 2984, 2936, 1647, 1316, 1287, 1154, 1132, 1051, 1028, 979 cm^−1^; HRMS (ESI-QTOF): calcd: 337.01578 [*M*+H]^+^; found: 337.0166; calcd: 358.99772 [*M*+Na]^+^; found: 358.9986;: calcd: 374.97166 [*M*+K]^+^; found: 374.9726; elemental analysis calcd (%) for C_13_H_15_Cl_2_O_4_P: C 46.31, H 4.48; found: C 46.39, H 4.41.

*Compound **25 b***: M.p. 86–89 °C; ^1^H NMR (400.27 MHz, CDCl_3_, 25 °C): *δ*=7.42–7.33 (m, 5 H; CH_arom_), 7.13 (d, ^3^*J*(H,P)=6.2 Hz, 1 H; =CH=), 5.64 (ddd, ^3^*J*(H,H)=2.8, 12.4, ^3^*J*(H,P)=4.6 Hz, 1 H; OCH), 2.27 (ABX system, A-part: dd, ^2^*J*(H,H)=15.3, ^3^*J*(H,H)*=*12.4 Hz, B-part: td, ^2^*J*(H,H)=15.3, ^3^*J*(H,H)*=*2.8 Hz, 2 H; CH_2_), 1.66 (s, 3 H; CH_3_), 1.54 ppm (d, *J*(P,H)=1.0 Hz, 3 H, CH_3_); ^13^C NMR (100.62 MHz, CDCl_3_, 25 °C): *δ*=138.5 (d, ^3^*J*(C,P)=6.3 Hz, C_arom_), 134.5 (d, ^2^*J*(C,P)=3.8 Hz, =HC=), 129.2 (s, CH_arom_), 129.1 (s, CH_arom_), 126.0 (s, CH_arom_), 113.0 (d, ^3^*J*(C,P)=14.9 Hz, =CCl_2_), 84.8 (d, ^2^*J*(C,P)=7.3 Hz, C-O), 79.6 (d, ^2^*J*(C,P)=6.2 Hz, O-CH), 44.1 (d, ^3^*J*(C,P)=9.8 Hz, CH_2_), 30.6 (d, ^3^*J*=3.5 Hz, CH_3_), 27.6 ppm (d, ^3^*J*(C,P)=4.0 Hz, CH_3_); ^31^P NMR (162.03 MHz, CDCl_3_, 25 °C): *δ*=−12.18 ppm (s); IR (ATR): $\tilde \nu $
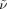
=3069, 2982, 2936, 1646, 1311, 1286, 1157, 1133, 1055, 1035, 977 cm^−1^; HRMS (ESI-QTOF): calcd: 337.01578 [*M*+H]^+^; found: 337.0170; calcd: 358.99772 [*M*+Na]^+^; found: 358.9990; calcd: 374.97166 [*M*+K]^+^; found: 374.9729.
