# Supplementary material for: The Stereochemical Course of the α-Hydroxyphosphonate–Phosphate Rearrangement
Source: Chemistry. 2015 Jun 8;21(28):10200–6. doi: 10.1002/chem.201406661 (PMC4515106; doi:10.1002/chem.201406661)
Supplement: Supplementary file 1 [file chem0021-10200-sd1.pdf]

CHEMISTRY
A European Journal

Supporting Information

The Stereochemical Course of the α-Hydroxyphosphonate–Phosphate Rearrangement

Katharina Pallitsch,[a] Alexander Roller,[b] and Friedrich Hammerschmidt*[a]

chem_201406661_sm_miscellaneous_information.pdf

<u>Overview of compounds:</u>

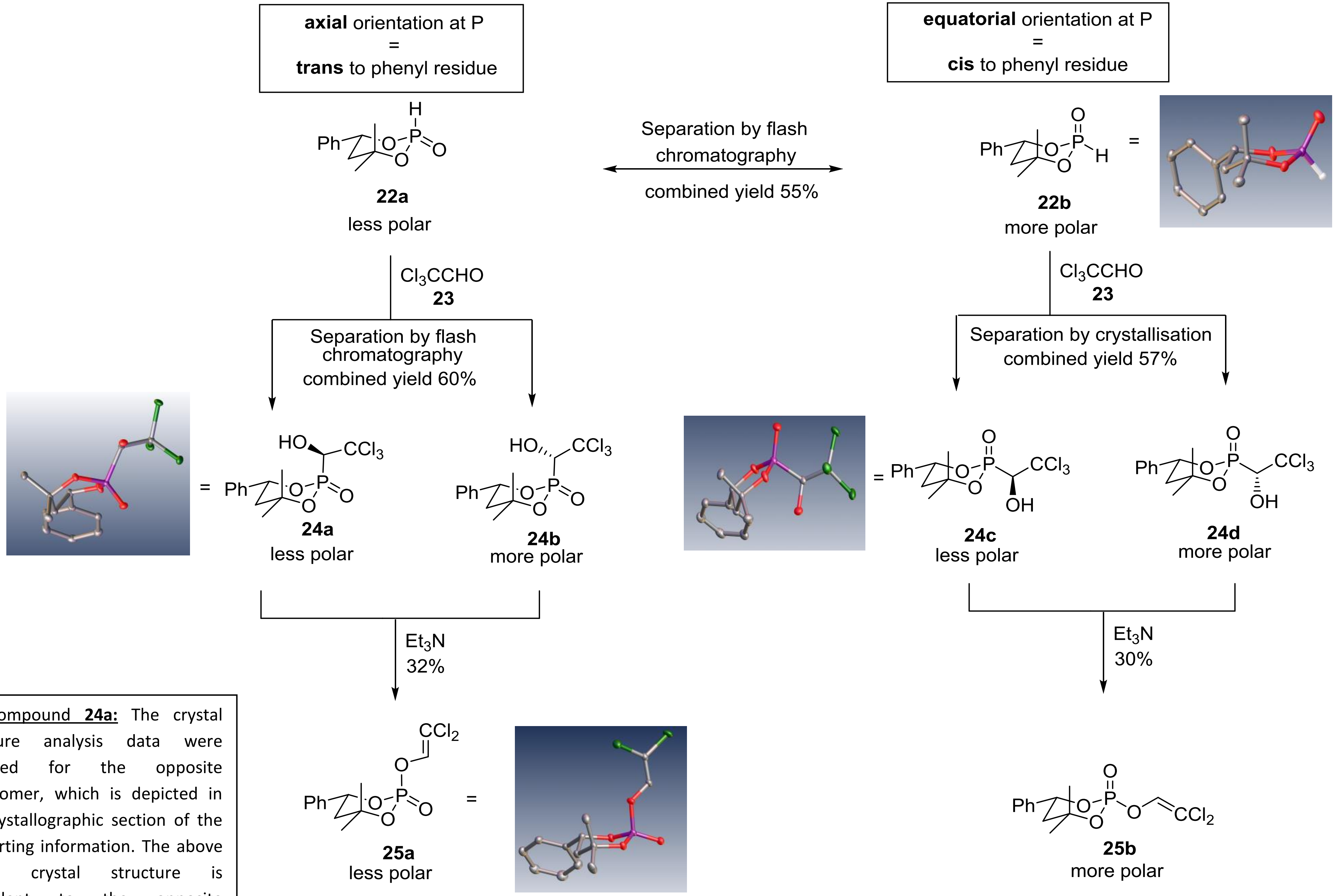

<u>For compound **24a:**</u> The crystal structure analysis data were obtained for the opposite enantiomer, which is depicted in the crystallographic section of the Supporting information. The above shown crystal structure is equivalent to the opposite enantiomer for graphical reasons.

^{31}P NMR kinetic study oft he rearrangement of **24a**:

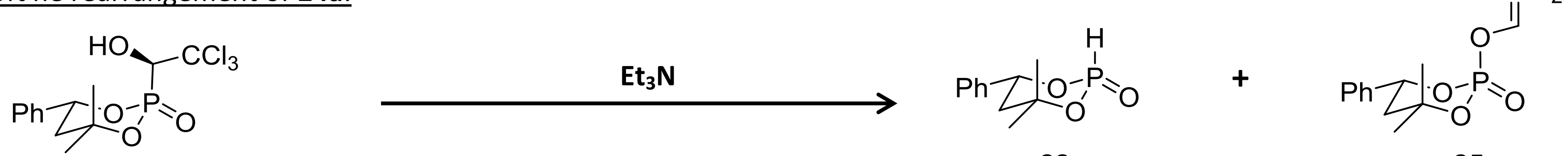

24a **9.38 ppm** → **22a** **-1.40 ppm** + **25a** **-11.76 ppm**

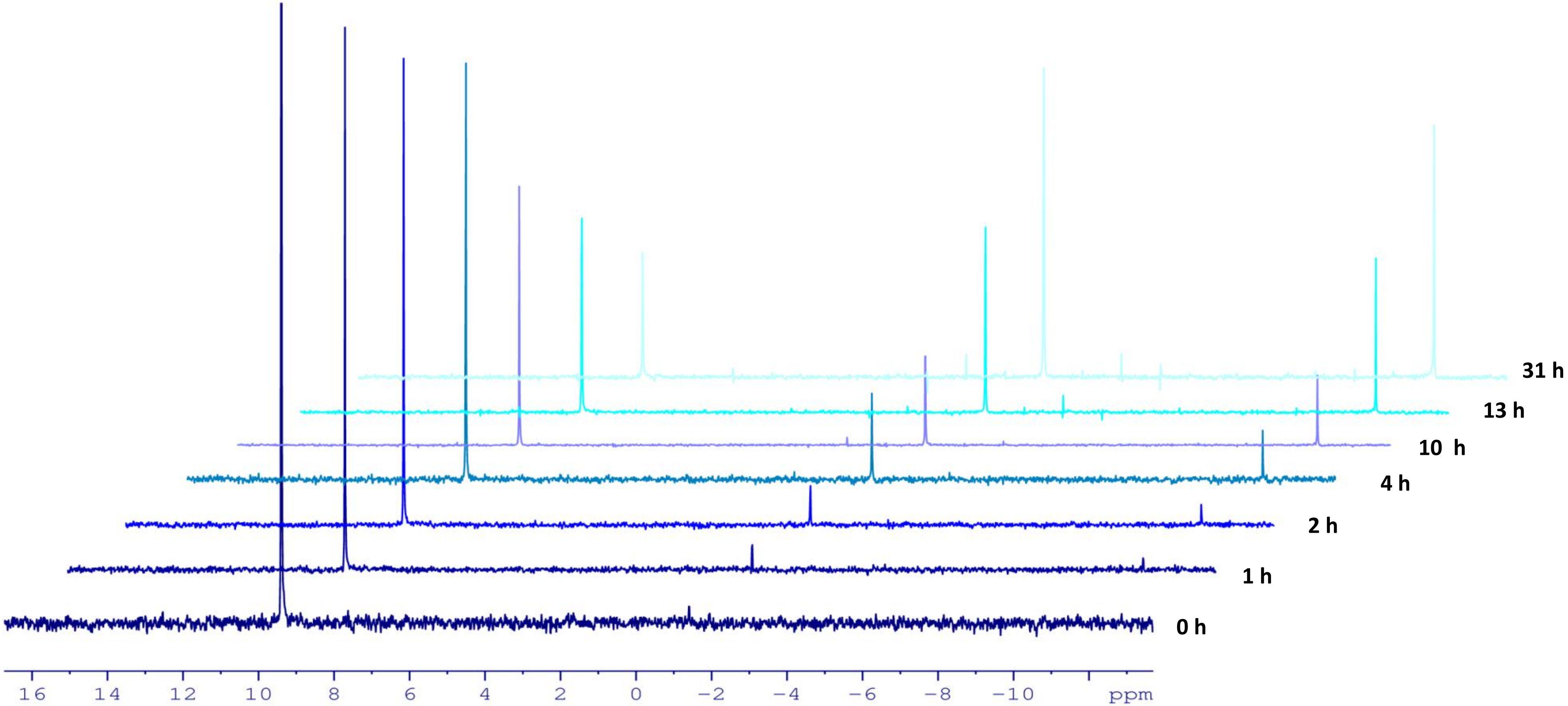

Representative ^{31}P NMR kinetics of the rearrangement of **24a** over 31 hours to give the desired phosphate **25a** and phosphite **22a** as side product. No diastereomeric hydroxyphosphonate, phosphate or phosphite was observed at any time during the course of the reaction. Deviations in the shifts (compared to the purified compounds) are due to the presence of Et_3N in the reaction mixture.

<u>^{1}H and ^{13}C NMR spectra of all compounds:</u>

All assignments are given in the “Experimental part” of the publication. The first spectrum shown of each series is the full ^{1}H-spectrum. Expansions are depicted where they were regarded as necessary. Then the ^{13}C spectrum is depicted in the same manner. The x-axes is in ppm, while the peak labels are in Hz for all given spectra. Structures are always given on top of the ^{1}H full spectrum. Integrals are denoted below the x-axes and the integration range is marked.. The numbering of the spectra is in accordance with the numbering of substances in the main text.

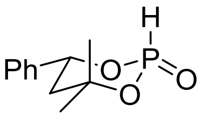

22a

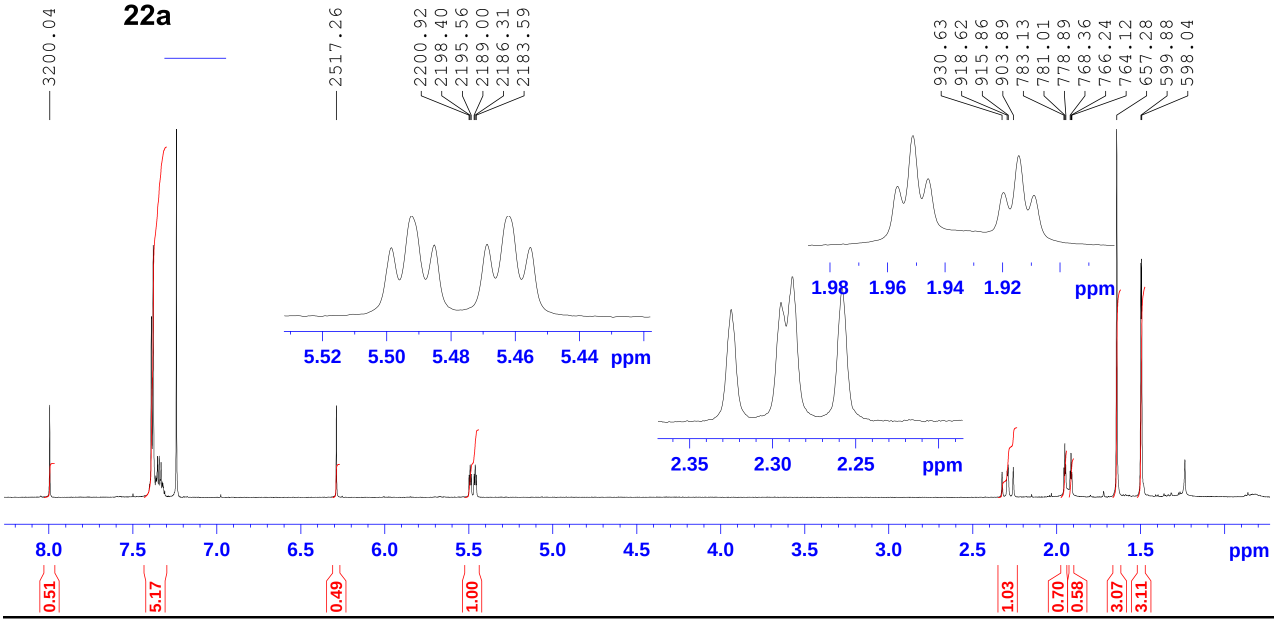

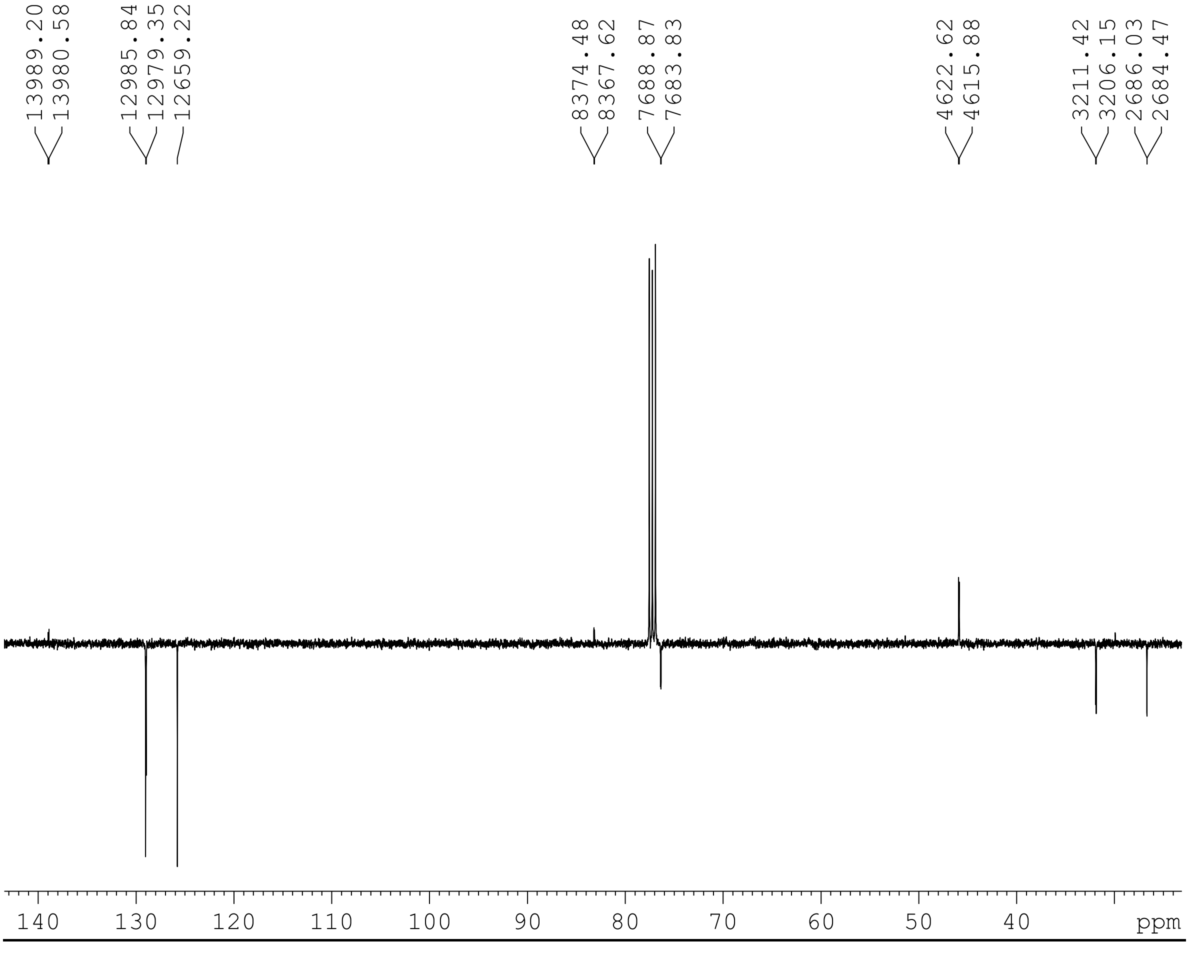

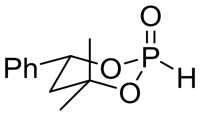

22b

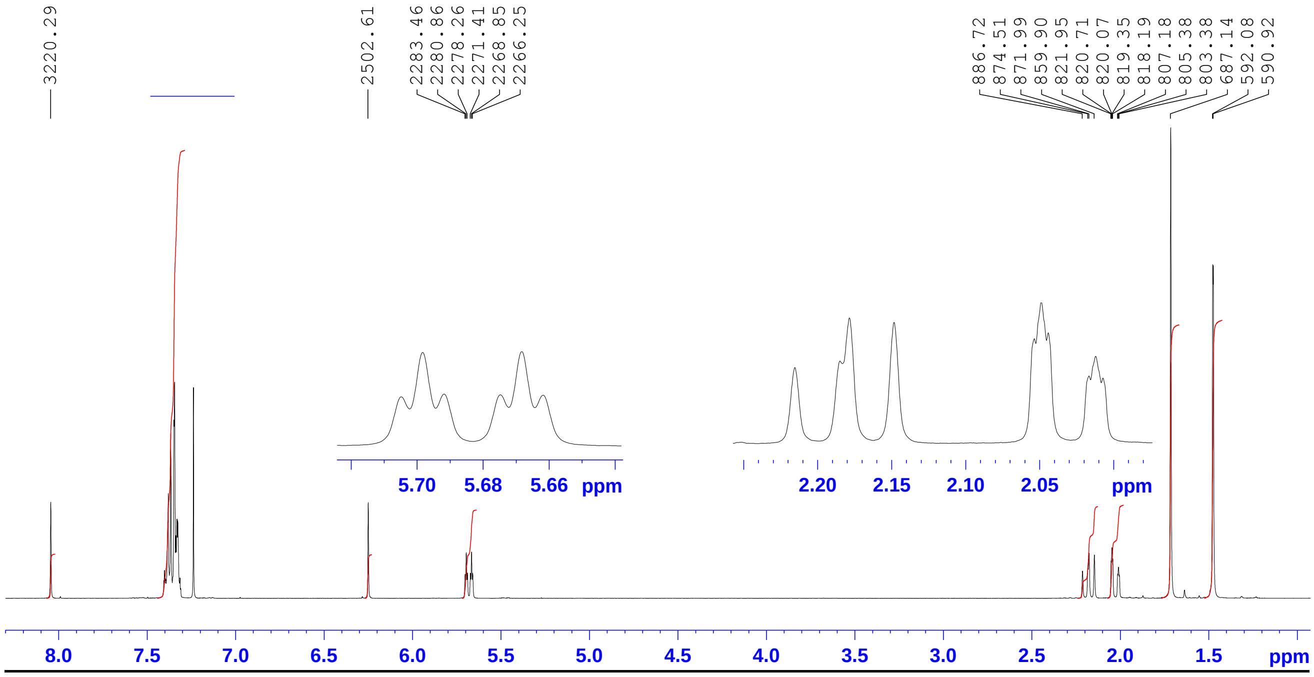
3220.29
2502.61
2283.46
2280.86
2278.26
2271.41
2268.85
2266.25
886.72
874.51
871.99
859.90
821.95
820.71
820.07
819.35
818.19
807.18
805.38
803.38
687.14
592.08
590.92
5.70 5.68 5.66 ppm
2.20 2.15 2.10 2.05 ppm
8.0 7.5 7.0 6.5 6.0 5.5 5.0 4.5 4.0 3.5 3.0 2.5 2.0 1.5 ppm

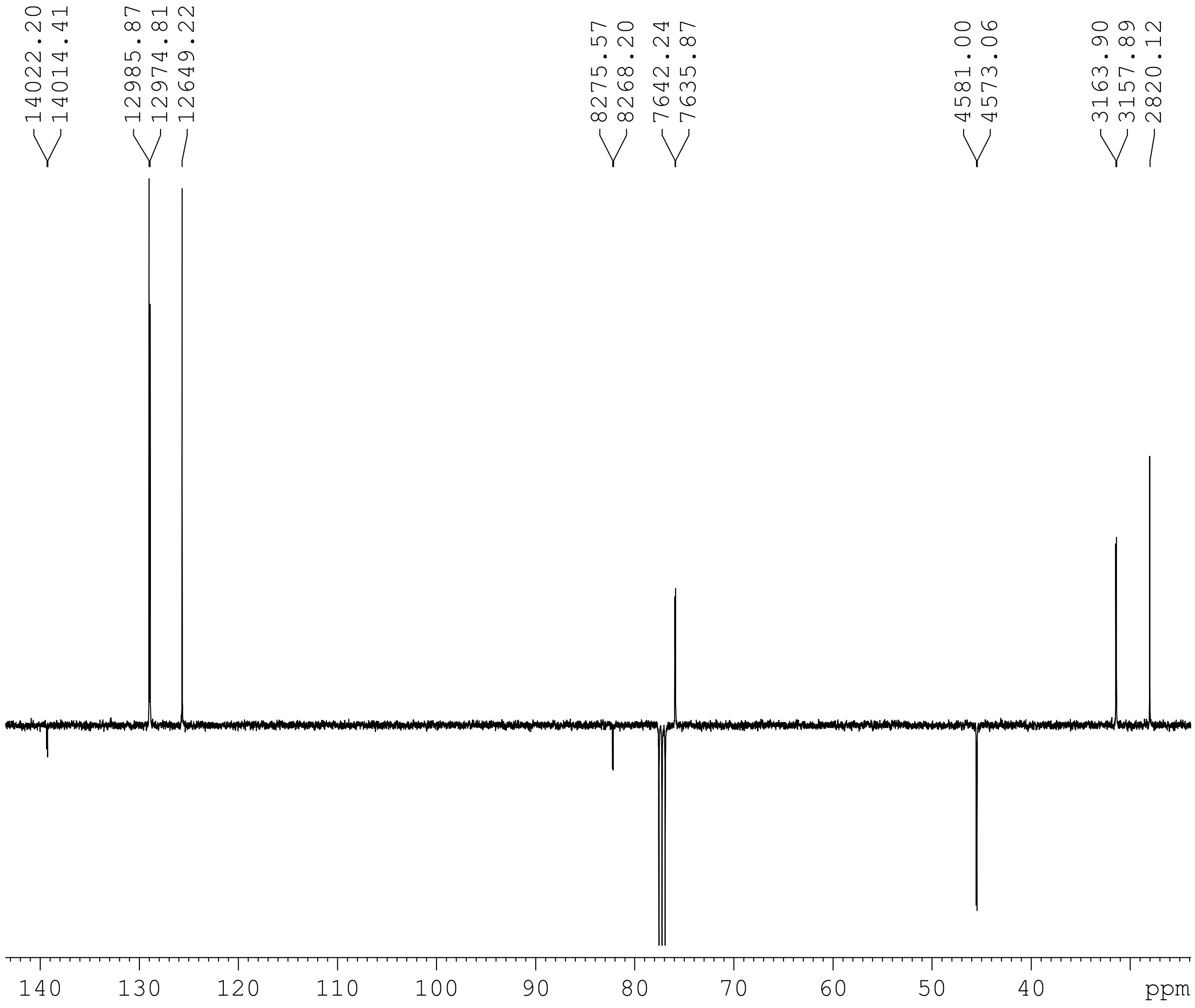
14022.20
14014.41
12985.87
12974.81
12649.22
8275.57
8268.20
7642.24
7635.87
4581.00
4573.06
3163.90
3157.89
2820.12
140 130 120 110 100 90 80 70 60 50 40 ppm

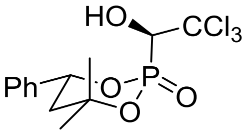
HO
CCl3
Ph
O
P
O
O

24a

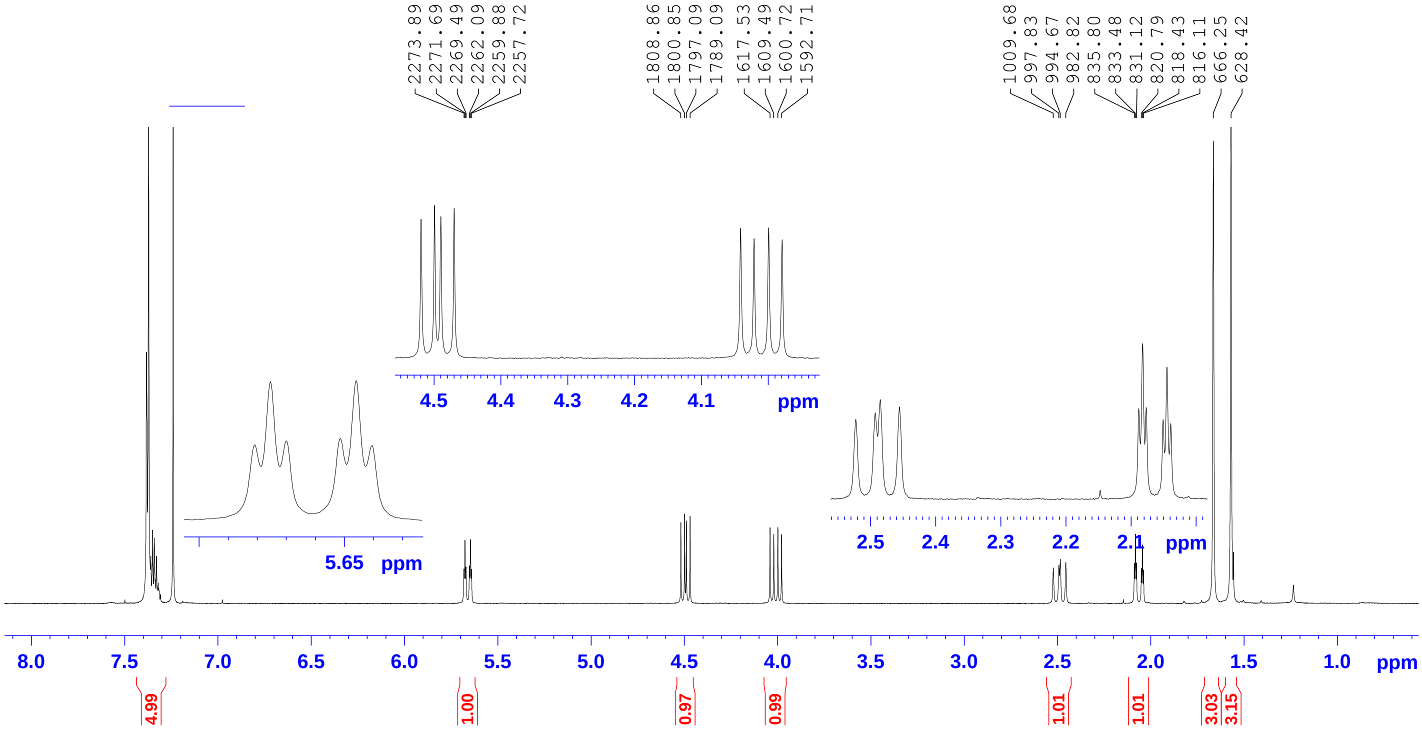
2273.89
2271.69
2269.49
2262.09
2259.88
2257.72
1808.86
1800.85
1797.09
1789.09
1617.53
1609.49
1600.72
1592.71
1009.68
997.83
994.67
982.82
835.80
833.48
831.12
820.79
818.43
816.11
666.25
628.42
5.65 ppm
4.5 4.4 4.3 4.2 4.1 ppm
2.5 2.4 2.3 2.2 2.1 ppm
8.0 7.5 7.0 6.5 6.0 5.5 5.0 4.5 4.0 3.5 3.0 2.5 2.0 1.5 1.0 ppm
4.99
1.00
0.97
0.99
1.01
1.01
3.03
3.15

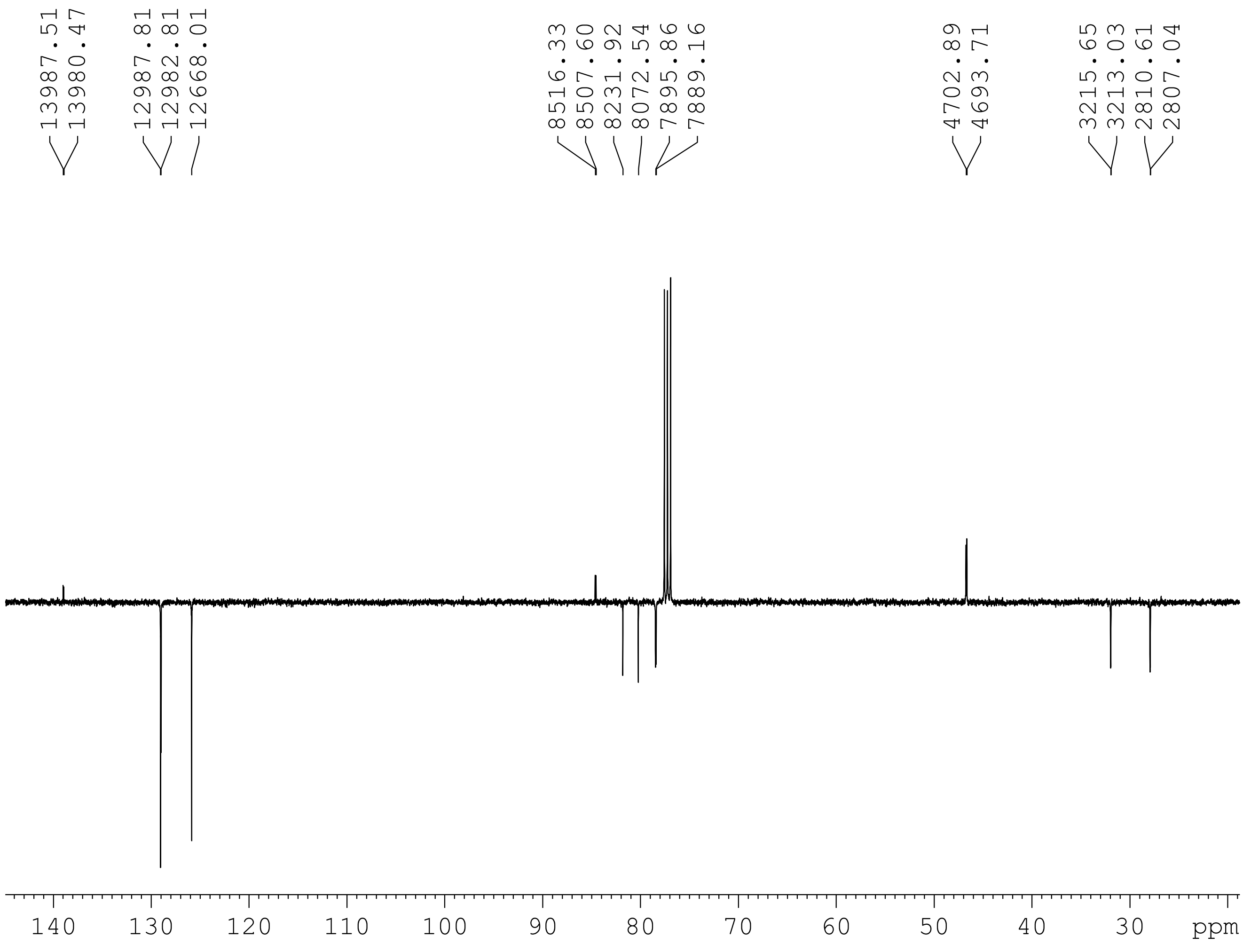
13987.51
13980.47
12987.81
12982.81
12668.01
8516.33
8507.60
8231.92
8072.54
7895.86
7889.16
4702.89
4693.71
3215.65
3213.03
2810.61
2807.04
140 130 120 110 100 90 80 70 60 50 40 30 ppm

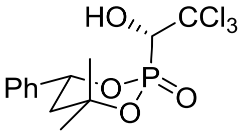
HO
CCl3
Ph
O
P
O
O

24b

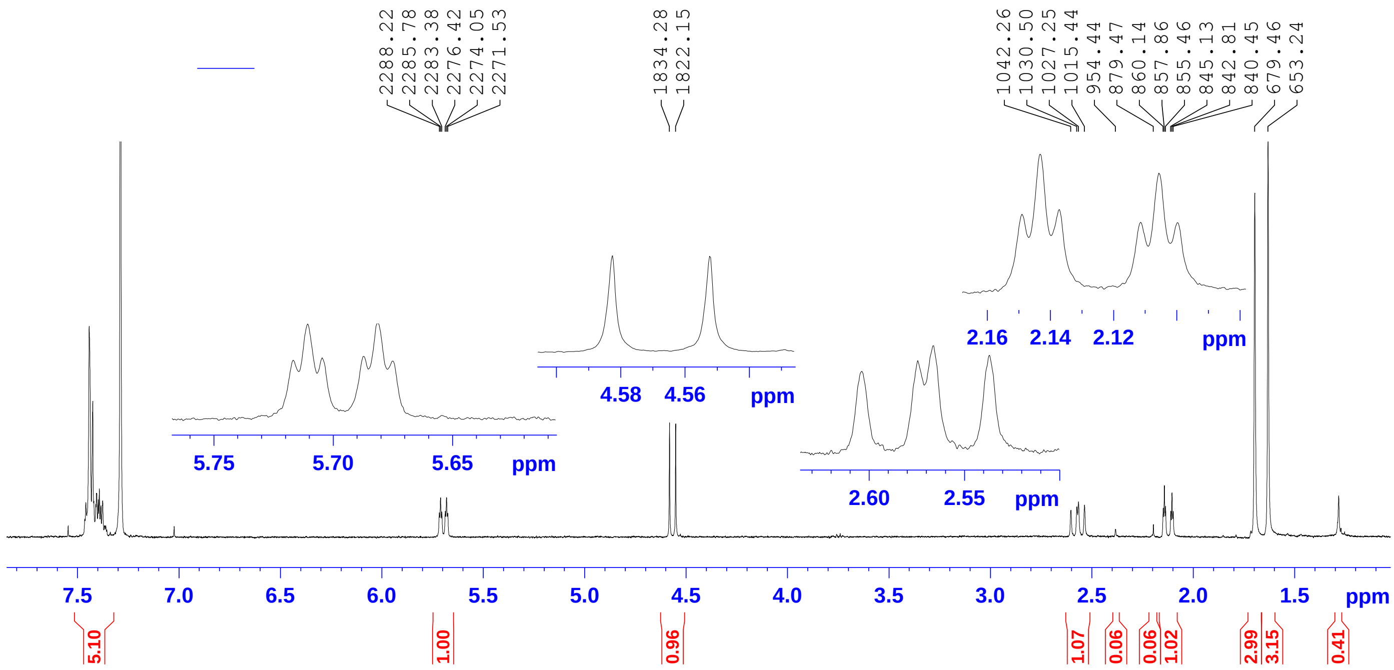
2288.22
2285.78
2283.38
2276.42
2274.05
2271.53
1834.28
1822.15
1042.26
1030.50
1027.25
1015.44
954.44
879.47
860.14
857.86
855.46
845.13
842.81
840.45
679.46
653.24
5.75 5.70 5.65 ppm
4.58 4.56 ppm
2.60 2.55 ppm
2.16 2.14 2.12 ppm
7.5 7.0 6.5 6.0 5.5 5.0 4.5 4.0 3.5 3.0 2.5 2.0 1.5 ppm
5.10
1.00
0.96
1.07
0.06
0.06
1.02
2.99
3.15
0.41

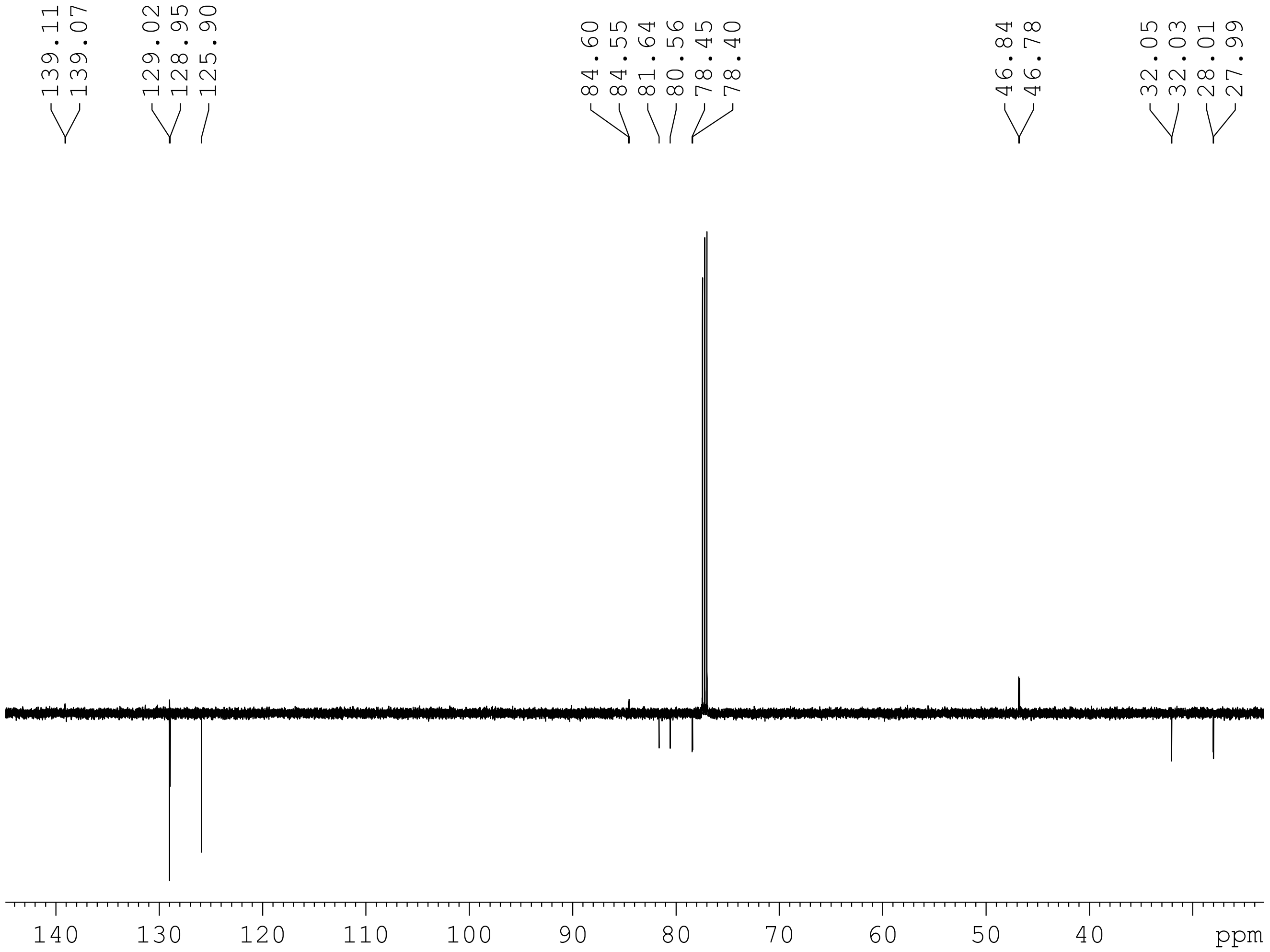
139.11
139.07
129.02
128.95
125.90
84.60
84.55
81.64
80.56
78.45
78.40
46.84
46.78
32.05
32.03
28.01
27.99
140 130 120 110 100 90 80 70 60 50 40 ppm

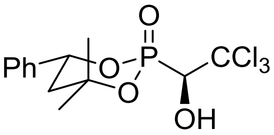
O
P
Ph
O
O
CCl3
OH

24c

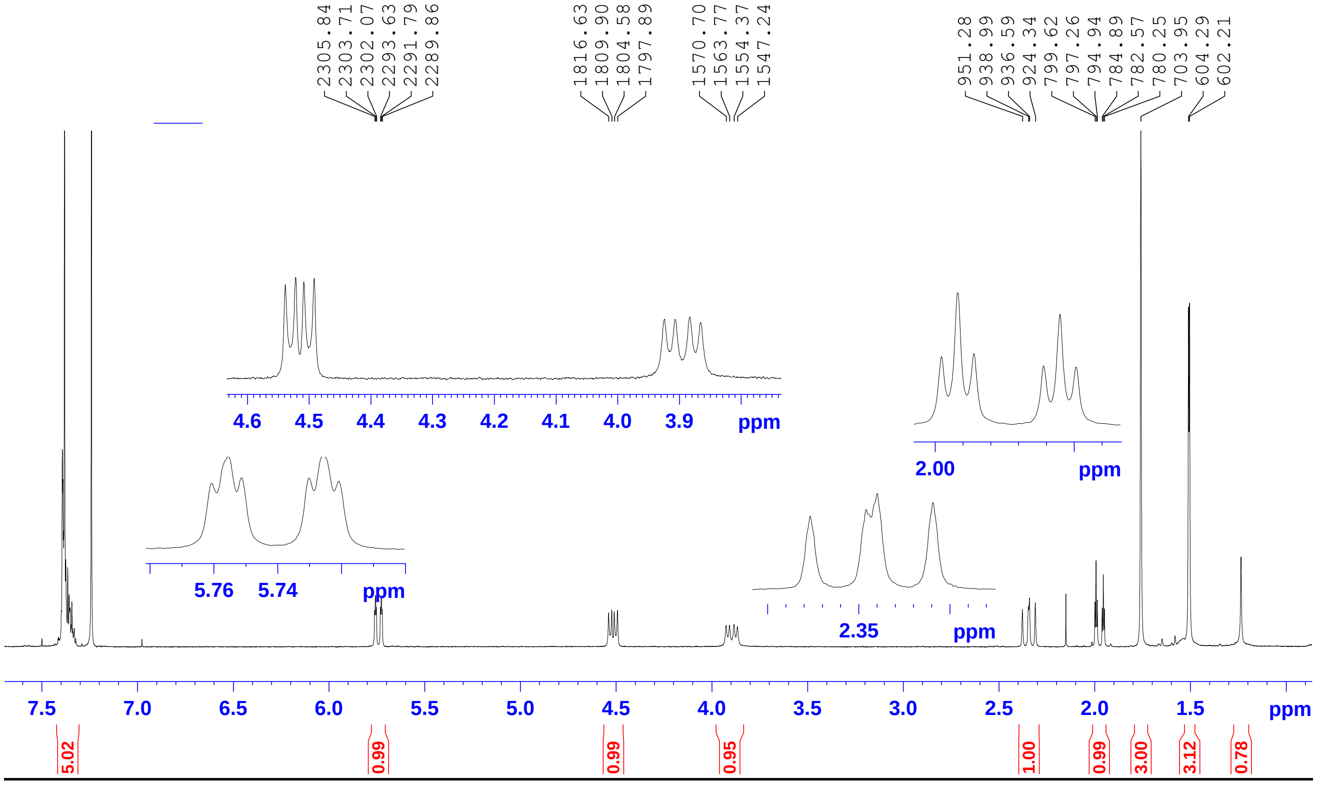
2305.84
2303.71
2302.07
2293.63
2291.79
2289.86
1816.63
1809.90
1804.58
1797.89
1570.70
1563.77
1554.37
1547.24
951.28
938.99
936.59
924.34
799.62
797.26
794.94
784.89
782.57
780.25
703.95
604.29
602.21
4.6 4.5 4.4 4.3 4.2 4.1 4.0 3.9 ppm
2.00 ppm
5.76 5.74 ppm
2.35 ppm
7.5 7.0 6.5 6.0 5.5 5.0 4.5 4.0 3.5 3.0 2.5 2.0 1.5 ppm
5.02
0.99
0.99
0.95
1.00
0.99
3.00
3.12
0.78

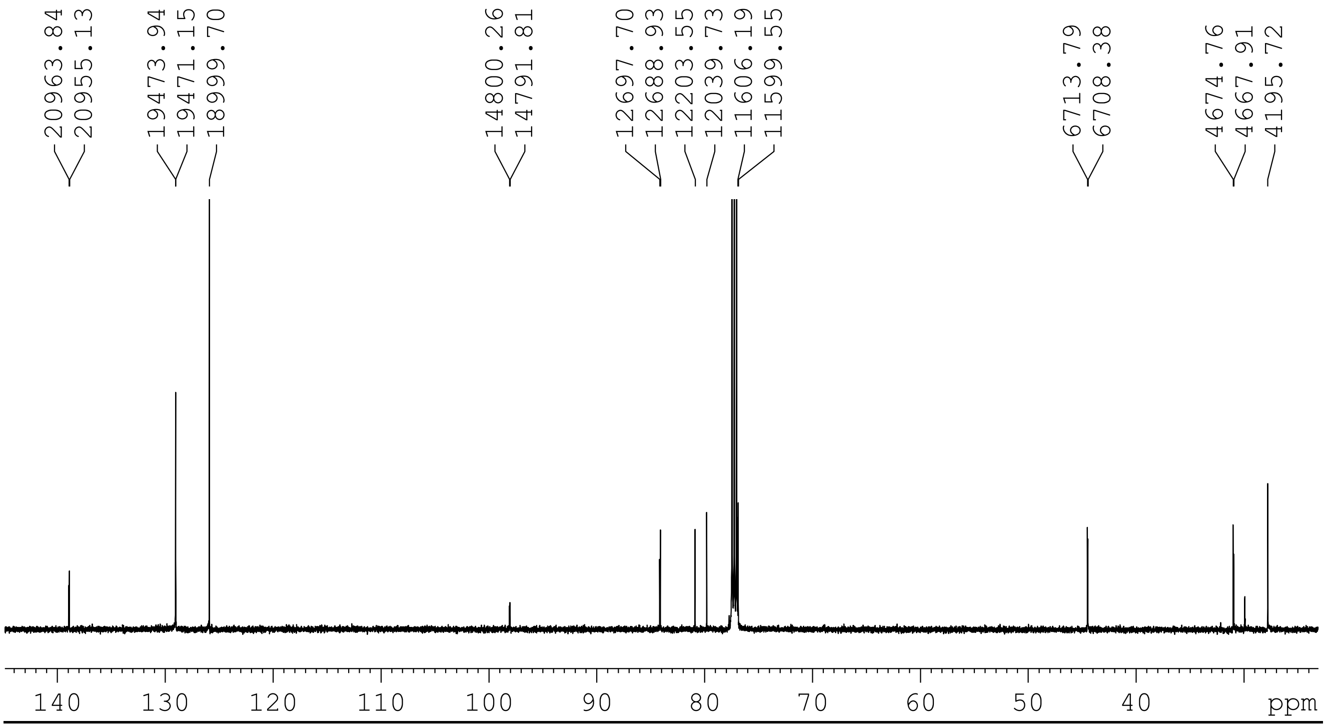
20963.84
20955.13
19473.94
19471.15
18999.70
14800.26
14791.81
12697.70
12688.93
12203.55
12039.73
11606.19
11599.55
6713.79
6708.38
4674.76
4667.91
4195.72
140 130 120 110 100 90 80 70 60 50 40 ppm

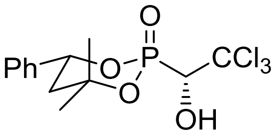
Ph
O
P
O
O
CCl3
OH

24d

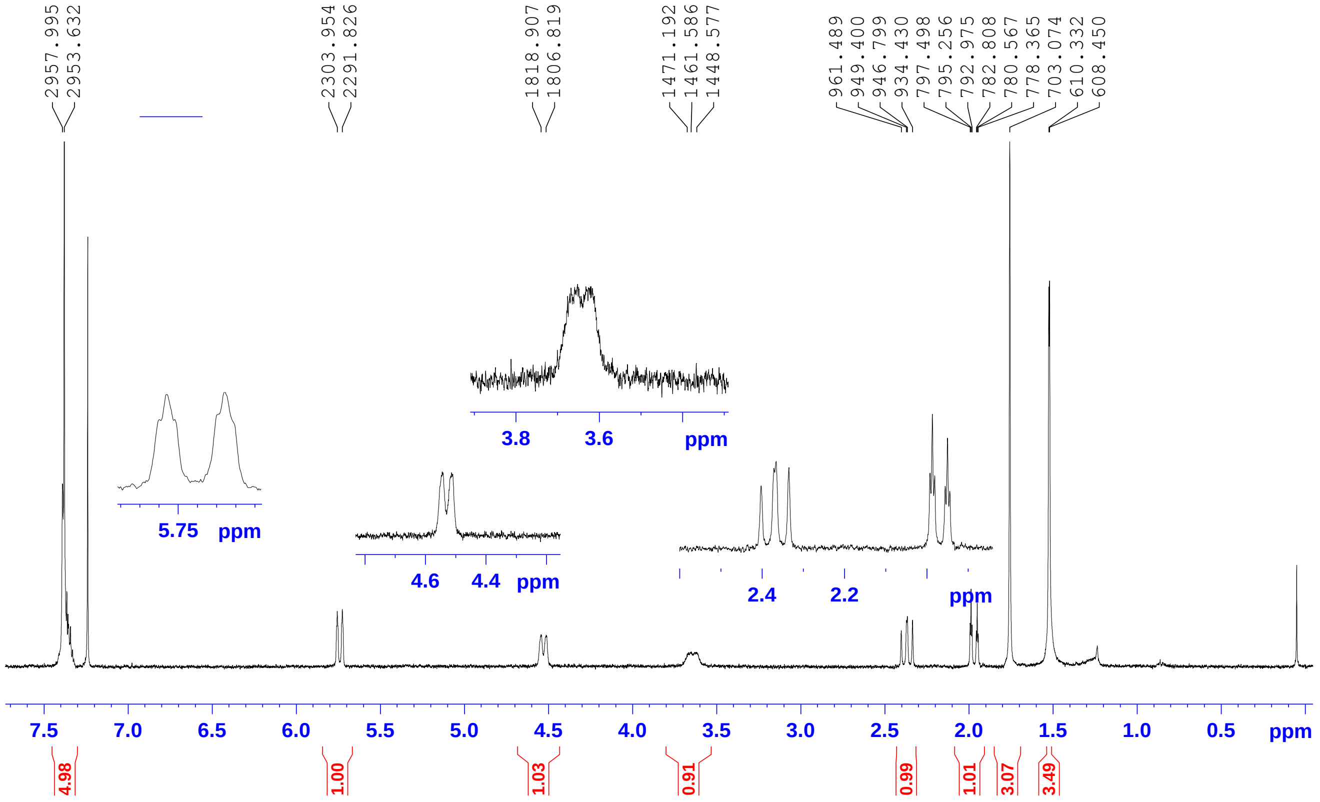
2957.995
2953.632
2303.954
2291.826
1818.907
1806.819
1471.192
1461.586
1448.577
961.489
949.400
946.799
934.430
797.498
795.256
792.975
782.808
780.567
778.365
703.074
610.332
608.450
5.75 ppm
3.8 3.6 ppm
4.6 4.4 ppm
2.4 2.2 ppm
7.5 7.0 6.5 6.0 5.5 5.0 4.5 4.0 3.5 3.0 2.5 2.0 1.5 1.0 0.5 ppm
4.98
1.00
1.03
0.91
0.99
1.01
3.07
3.49

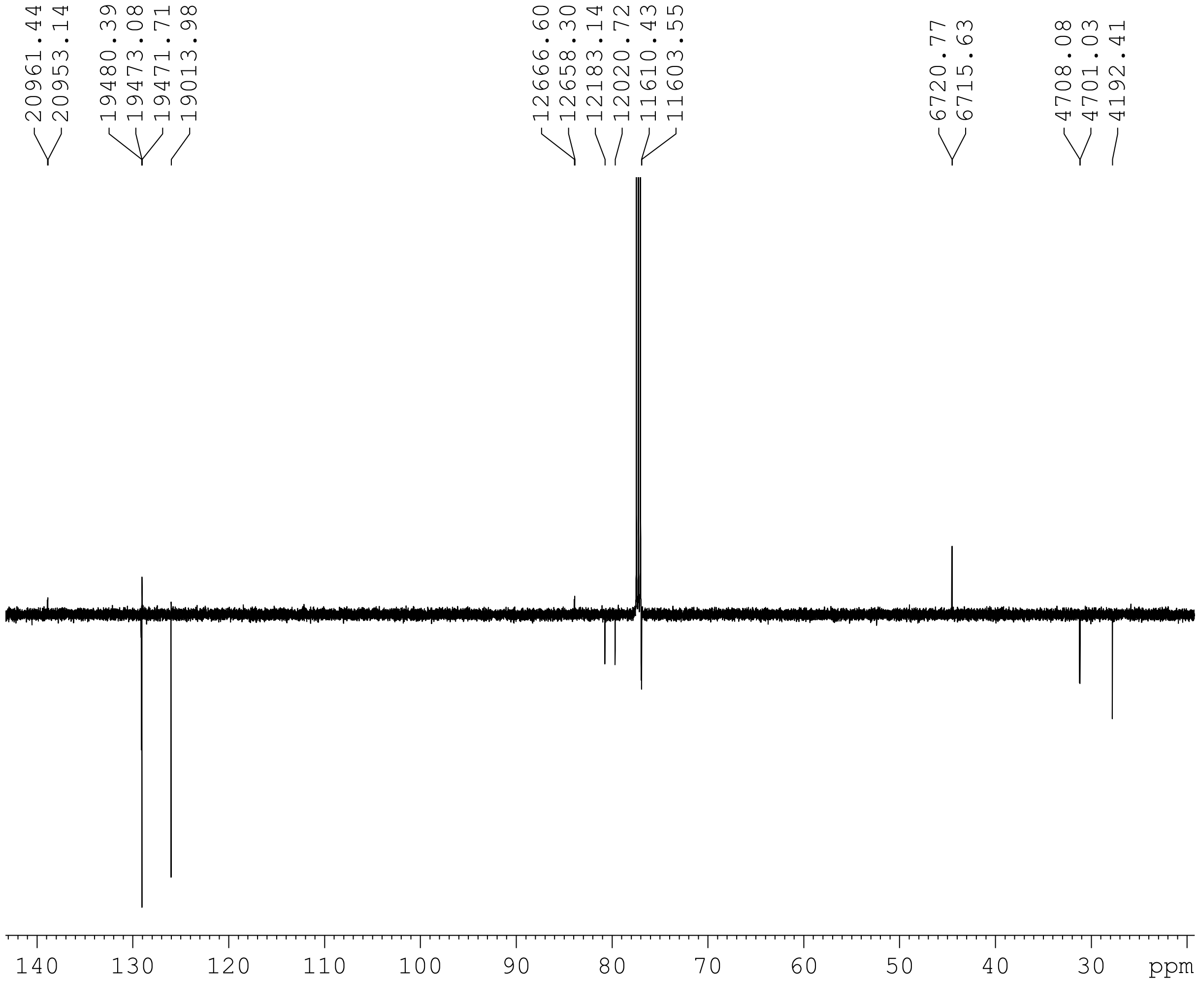
20961.44
20953.14
19480.39
19473.08
19471.71
19013.98
12666.60
12658.30
12183.14
12020.72
11610.43
11603.55
6720.77
6715.63
4708.08
4701.03
4192.41
140 130 120 110 100 90 80 70 60 50 40 30 ppm

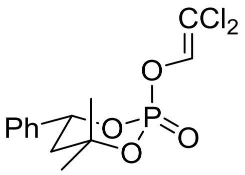
CCl2
O
P
O
O
Ph

25a

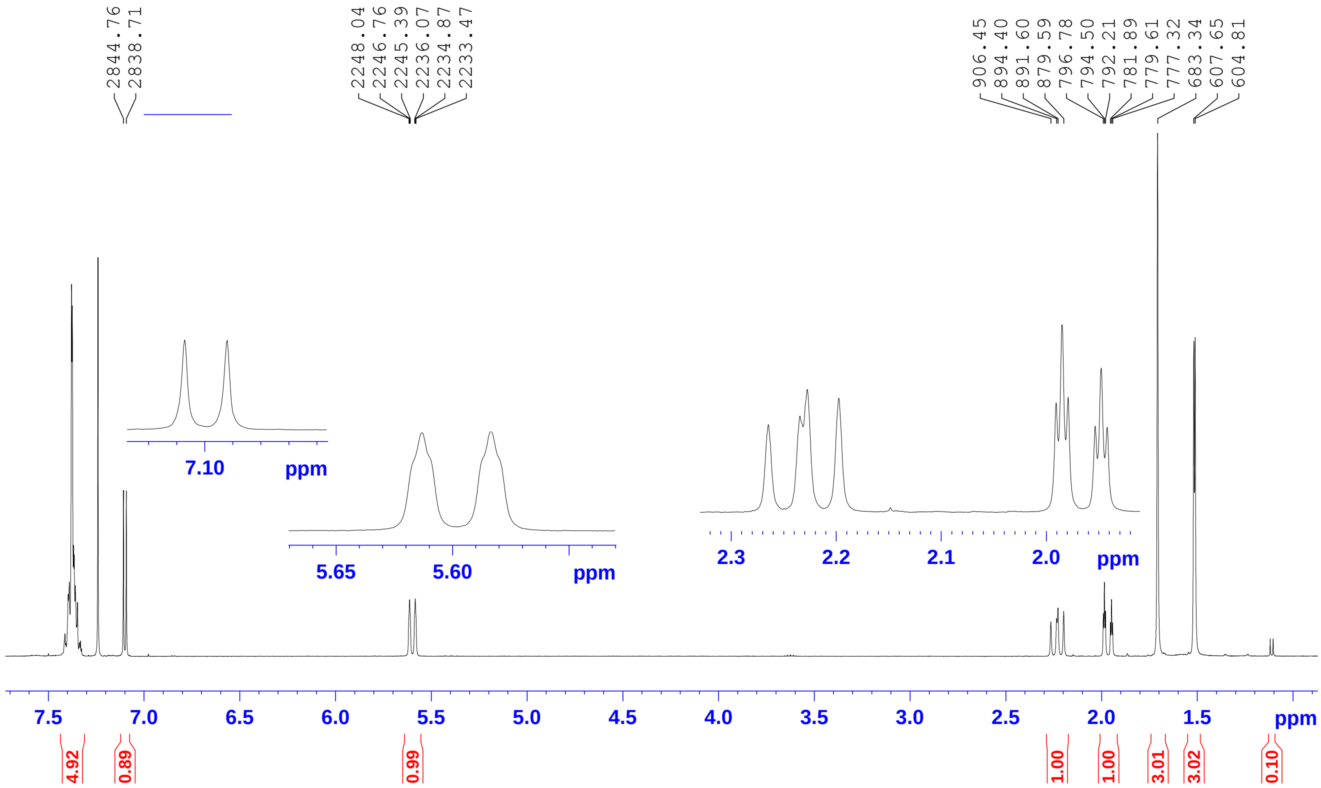
2844.76
2838.71
2248.04
2246.76
2245.39
2236.07
2234.87
2233.47
906.45
894.40
891.60
879.59
796.78
794.50
792.21
781.89
779.61
777.32
683.34
607.65
604.81
7.10
ppm
5.65
5.60
ppm
2.3
2.2
2.1
2.0
ppm
7.5
7.0
6.5
6.0
5.5
5.0
4.5
4.0
3.5
3.0
2.5
2.0
1.5
ppm
4.92
0.89
0.99
1.00
1.00
3.01
3.02
0.10

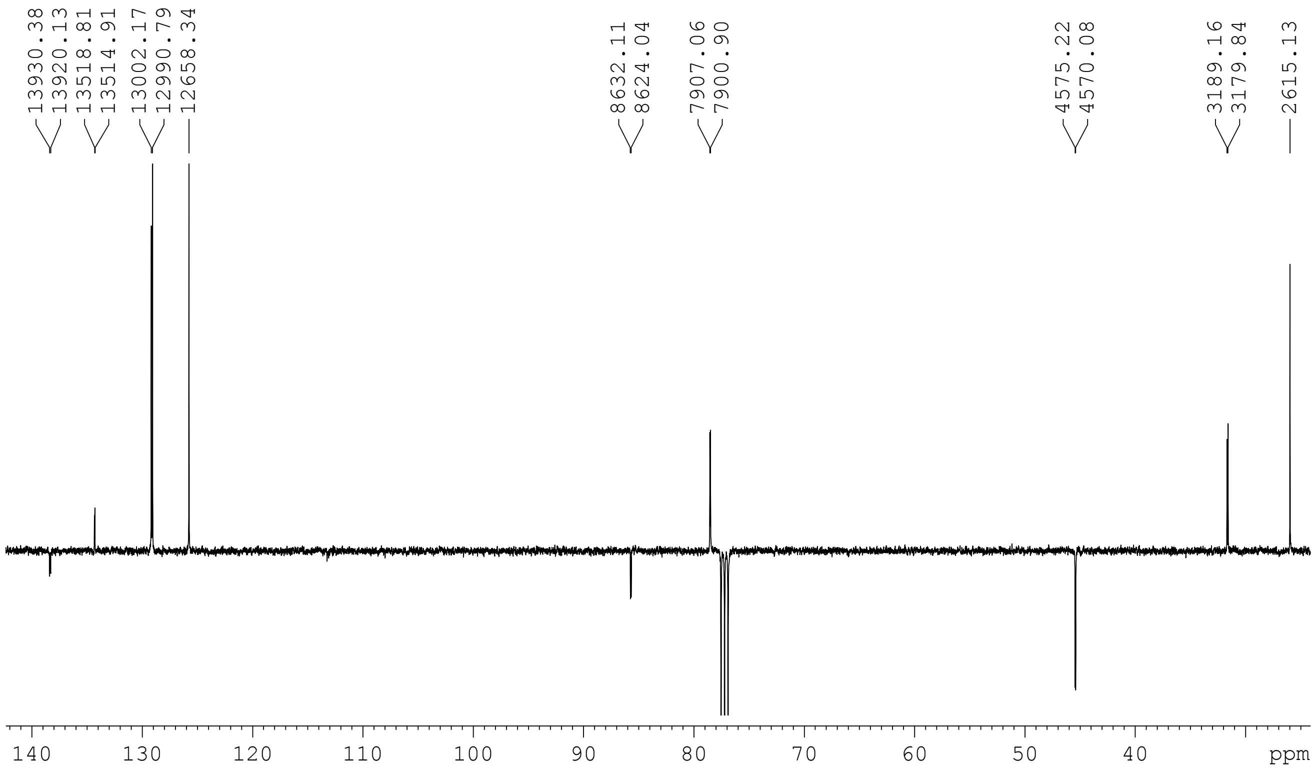
13930.38
13920.13
13518.81
13514.91
13002.17
12990.79
12658.34
8632.11
8624.04
7907.06
7900.90
4575.22
4570.08
3189.16
3179.84
2615.13
140
130
120
110
100
90
80
70
60
50
40
ppm

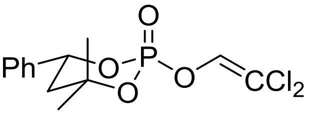
Ph
O
P
O
O
O
CCl2

25b

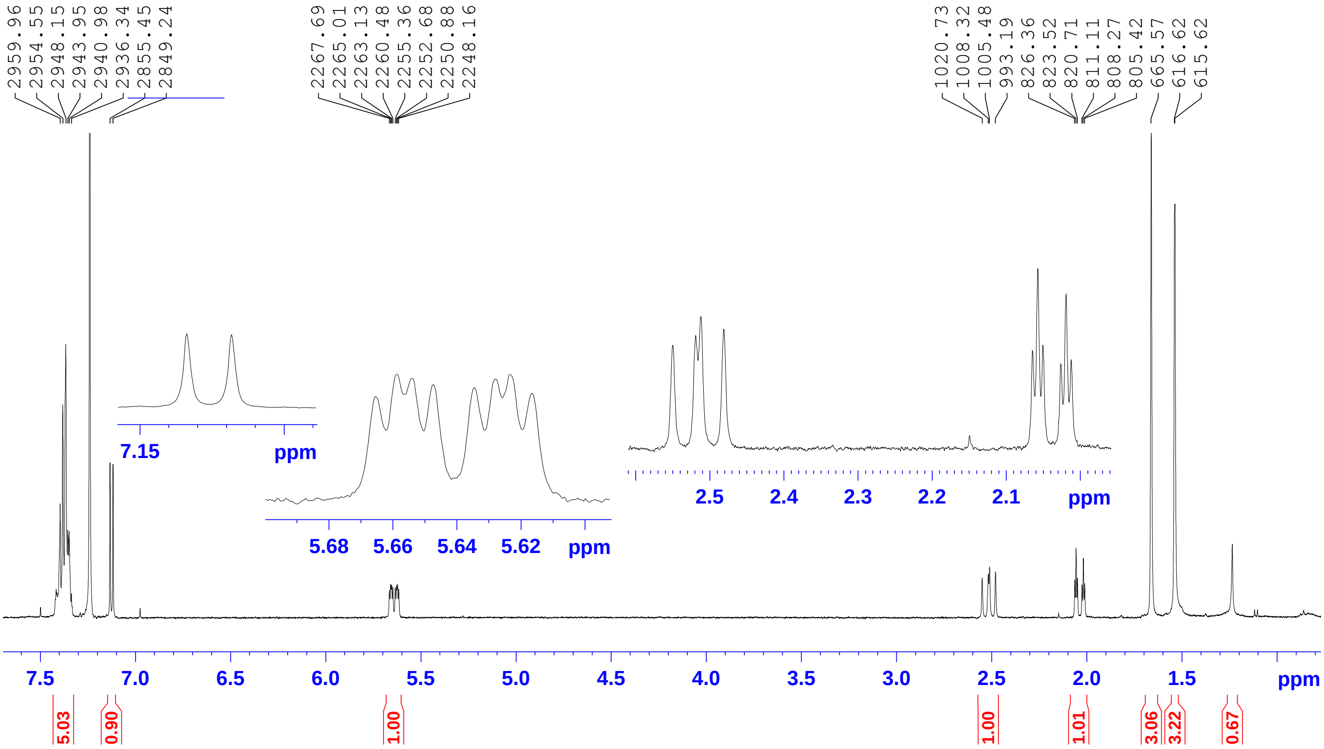
2959.96
2954.55
2948.15
2943.95
2940.98
2936.34
2855.45
2849.24
2267.69
2265.01
2263.13
2260.48
2255.36
2252.68
2250.88
2248.16
1020.73
1008.32
1005.48
993.19
826.36
823.52
820.71
811.11
808.27
805.42
665.57
616.62
615.62
7.15
ppm
5.68
5.66
5.64
5.62
ppm
2.5
2.4
2.3
2.2
2.1
ppm
7.5
7.0
6.5
6.0
5.5
5.0
4.5
4.0
3.5
3.0
2.5
2.0
1.5
ppm
5.03
0.90
1.00
1.00
1.01
3.06
3.22
0.67

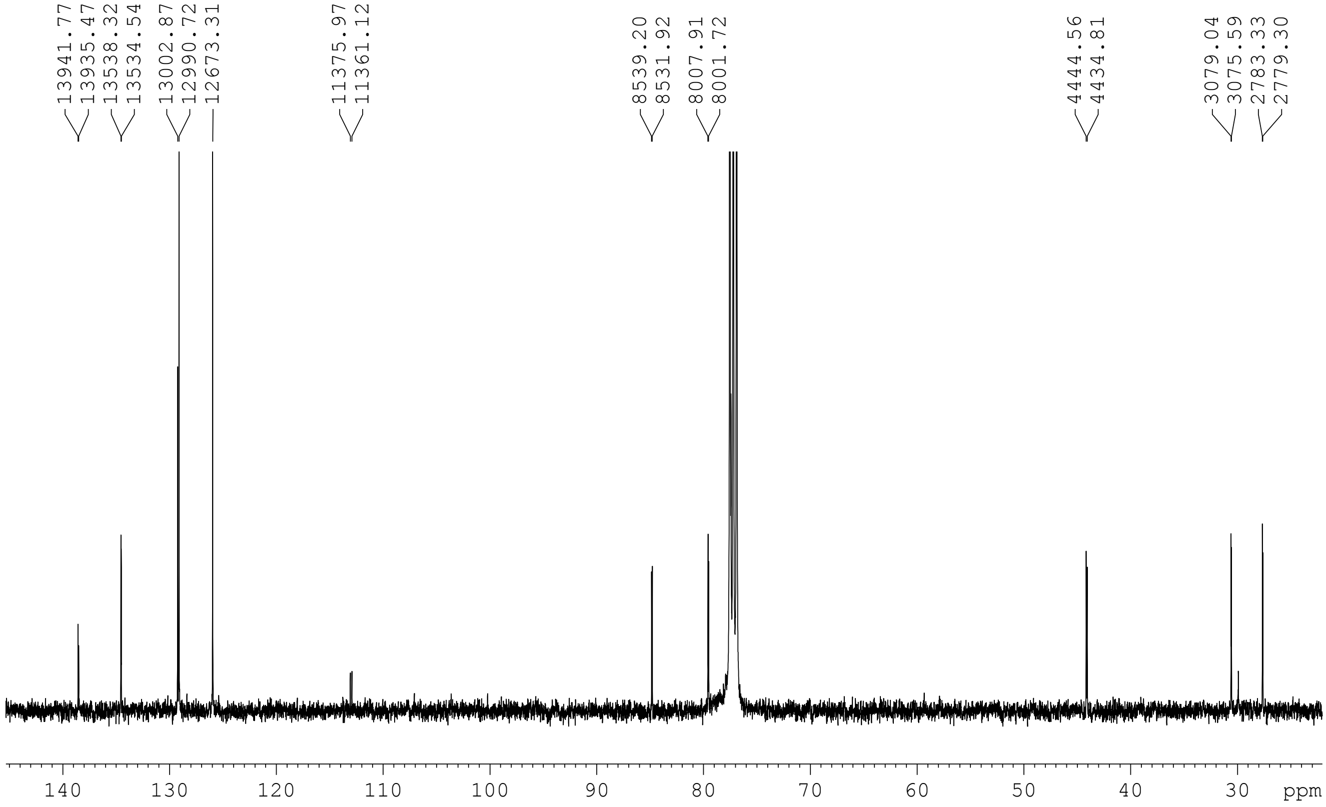
13941.77
13935.47
13538.32
13534.54
13002.87
12990.72
12673.31
11375.97
11361.12
8539.20
8531.92
8007.91
8001.72
4444.56
4434.81
3079.04
3075.59
2783.33
2779.30
140
130
120
110
100
90
80
70
60
50
40
30
ppm

X-ray crystallography:

The X-ray intensity data were measured on a Bruker Bruker D8-Venture diffractometer equipped with a multilayer monochromator, a Mo K/a INCOATEC micro focus sealed tube (λ = 0.71073 Å) and a Kryoflex II cooling device. The structures were solved by direct methods and refined by full-matrix least-squares techniques. Non-hydrogen atoms were refined with anisotropic displacement parameters. Hydrogen atoms were inserted in calculated positions and refined with a riding model. The following computer programs and hardware were used: Frame integration, *Bruker SAINT software package*[1] using a narrow-frame algorithm, Absorption correction, *SADABS*[2] , structure solution, *SHELXS-97*[3], refinement, *SHELXL-2013*[3], *OLEX2*[4], *SHELXLE*[5], molecular diagrams, *OLEX2*[4]. Crystal data, data collection parameters, and structure refinement details are given in Tables 1-9. Molecular Structures in "Ortep View" are discussed in Figures 1 - 4.

**Sample**	**T**	**Detector distance**	**Time/Frame**	**Frames collected**	**Frame Width**	**CCDC**
	[K]	[mm]	[s]		[°]	
22b	100 (2)	35	64	1180	0.4	1040511
24a	100 (2)	35	32	561	0.4	1040512
24c	100 (2)	50	48	2535	0.4	1040513
25a	100 (2)	40	32	1692	0.4	1040514

Table 1. Experimental parameters and CCDC-CODE's.

1) Compound (22b) submitted for X-ray structure analysis

Compound name and number in manuscript for *Chemistry A European Journal*.: (2*R**,6*R**)-4,4-Dimethyl-6-phenyl-1,3,2-dioxaphosphinane-2-oxide (**22b**)

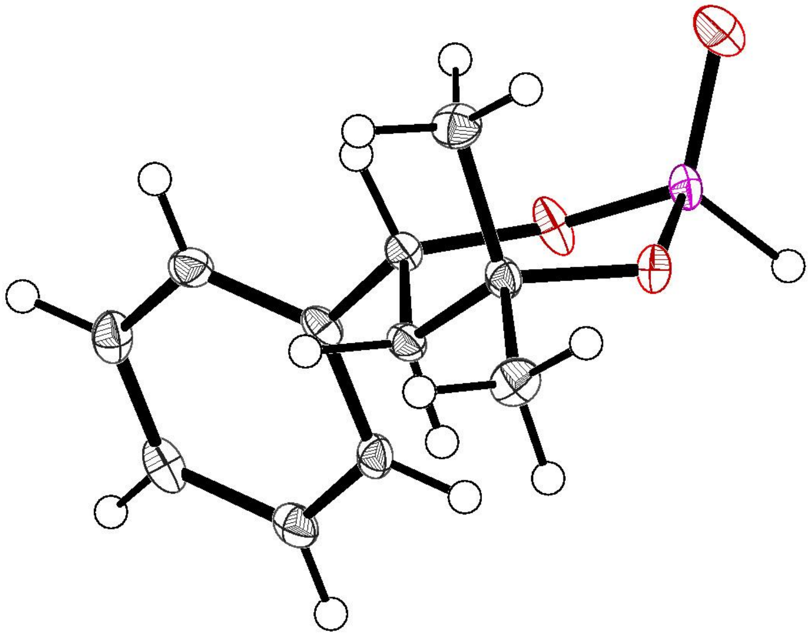

Figure 1. . Molecular structure of (**22b**), drawn with 50% displacement ellipsoids. The highlighted molecule corresponds to the asymmetric unit. Two different hydrogen bond distances were recorded: The intermolecular donor-acceptor (O-H··· O) distance, 2.64059(7) Å, and the intramolecular donor-acceptor (N-H··· O) distance, 2.95427(12) Å. Taking into account the corresponding angles of 173.29238 (19)° and 133.965 (2)° and following the classification of Jeffrey[6] it can be supposed that the intermolecular hydrogen bond is of moderate character and the intramolecular hydrogen bond is of weak character. The molecular structure in the space group $P2_12_12_1$ and the value of the FLACK parameter (Flack x = -0.01(4) by hole-in-one fit to all intensities) confirm, that in consilience with the synthetic path and the NMR results, (**22b**)crystallized in the correct chiral form.

**Chemical formula**	C11H15O3P	**Crystal system**	orthorhombic	
**Formula weight**	226.20	**Space group**	$P\ 2_1\ 2_1\ 2_1$	
**Temperature**	100(2) K	**Z**	4	
**Measurement method**	\Φ and \ω scans	**Volume**	1107.37(8) Å^3	
**Radiation**	Mo K\α	**Unit cell dimensions**	a = 6.0523(2) Å	α = 90°
**Wavelength**	0.71073 Å			
**Crystal size**	0.284 x 0.052 x 0.014 mm^3		b = 10.6398(5) Å	β = 90°
**Crystal habit**	clear colorless plate		c = 17.1965(7) Å	γ = 90°
**Density (calculated)**	1.357 g/cm^3	**Absorption coefficient**	0.232 mm^{-1}	
**Abs. correction Tmin**	0.6736	**Abs. correction Tmax**	0.7460	
**Abs. correction type**	multi-scan	**F(000)**	480.0 e$^-$	

Table 2. Sample and crystal data of (**22b**).

**Theta range for data collection**	2.369 to 30.062°		**Index ranges**	-8<=h<=8, -14<=k<=14 , -24<=l<=24
**Refinement method**	Least squares		**Function minimized**	Σ w(F_o^2 - F_c^2)^2
**Reflections number**	21776			
**Data / restraints / parameters**	3231 / 0 / 142		**Goodness-of-fit on F^2**	1.057
**Final R indices**	2759 data; I>2σ(I)	R1 = 0.0398, wR2 = 0.0760	all data (3231)	R1 = 0.0541, wR2 = 0.0797
**Weighting scheme**	w=1/[$\sigma^2(F_o^2)+(0.0346P)^2+0.2942P$]		**Largest diff. peak and hole**	0.319 and -0.375 eÅ^{-3}
	where P=($F_o^2+2F_c^2$)/3		**R.M.S. deviation from mean**	0.067 eÅ^{-3}

Table 3. Data collection and structure refinement of (**22b**).

2) Compound (24a) submitted for X-ray structure analysis

Compound name and number in manuscript for *Chemistry A European Journal*.: (2*S**,6*R**,1'*R**)-4,4-Dimethyl-6-phenyl-2-(2,2,2-trichloro-1-hydroxy-ethyl)-1,3,2-dioxaphosphinane- 2-oxide (**24a**).

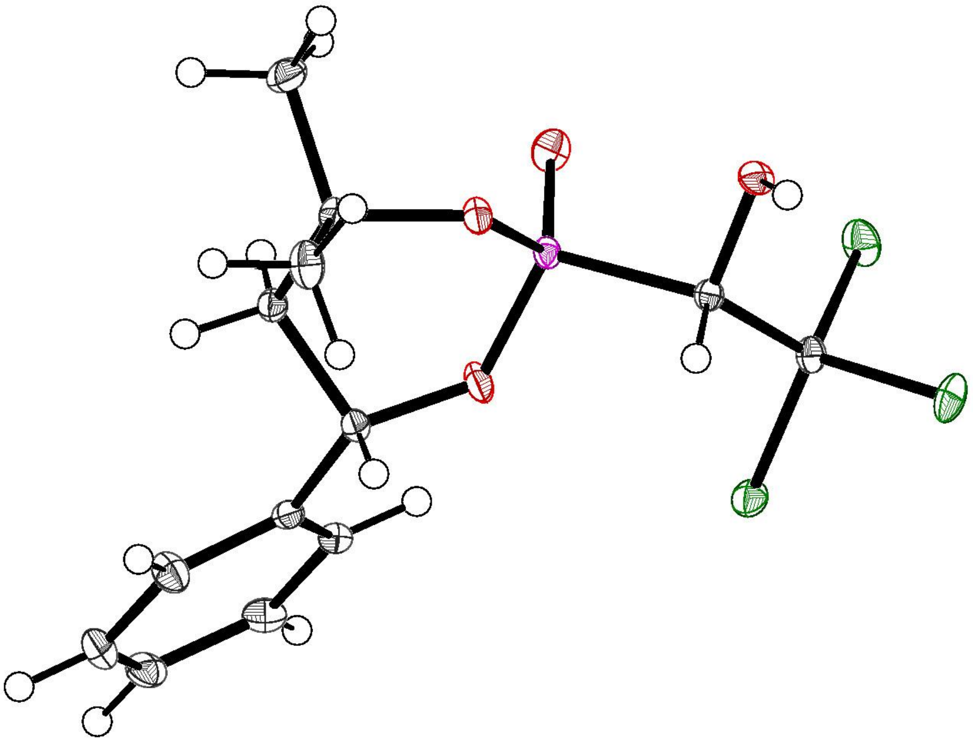

Figure 2. . Molecular structure of (**24a**), drawn with 50% displacement ellipsoids. The highlighted molecule corresponds to the asymmetric unit. One hydrogen bond distance is recorded. The intermolecular donor-acceptor (O-H··· O) distance is 2.73129(10) Å. Taking into account the corresponding angle of 161.4933 (6) ° and following the classification of Jeffrey[6] it can be supposed that this intermolecular hydrogen bond is of moderate character. The molecular structure in the space group $P2_12_12_1$ and the value of the FLACK parameter (Flack x = -0.009(15) by hole-in-one fit to all intensities) confirm, that in consilience with the synthetic path and the NMR results, (**24a**) crystallized in the correct chiral form.

**Chemical formula**	C13H16Cl3O4P	**Crystal system**	orthorhombic	
**Formula weight**	373.58	**Space group**	*P 2₁ 2₁ 2₁*	
**Temperature**	100(2) K	**Z**	4	
**Measurement method**	\Φ and \ω scans	**Volume**	1577.53(9) Å^3	
**Radiation**	Mo K\α	**Unit cell dimensions**	a = 5.6910(2) Å	α = 90°
**Wavelength**	0.71073 Å			
**Crystal size**	0.160 x 0.100 x 0.040 mm^3		b = 13.9766(4) Å	β = 90°
**Crystal habit**	clear colorless block		c = 19.8330(7) Å	γ = 90°
**Density (calculated)**	1.573 g/cm^3	**Absorption coefficient**	0.693 mm^{-1}	
**Abs. correction Tmin**	0.6791	**Abs. correction Tmax**	0.7460	
**Abs. correction type**	multi-scan	**F(000)**	768.0 e$^-$	

Table 4. Sample and crystal data of (**24a**).

**Theta range for data collection**	2.518to 30.018°		**Index ranges**	-7<=h<=7, -19<=k<=19 , -26<=l<=27
**Refinement method**	Least squares		**Function minimized**	Σ w(F$_o^2$ - F$_c^2$)^2
**Reflections number**	16285			
**Data / restraints / parameters**	4580 / 0 / 196		**Goodness-of-fit on F^2**	1.084
**Final R indices**	4434 data; I>2σ(I)	R1 = 0.0206, wR2 = 0.0478	all data (4580)	R1 = 0.0222, wR2 = 0.0487
**Weighting scheme**	w=1/[σ^2(F$_o^2$)+(0.0195P)^2+0.4257P]		**Largest diff. peak and hole**	0.283 and -0.206eÅ^{-3}
	where P=(F$_o^2$+2F$_c^2$)/3		**R.M.S. deviation from mean**	0.047 eÅ^{-3}

Table 5. Data collection and structure refinement of (**24a**).

3) Compound (24c) submitted for X-ray structure analysis

Compound name and number in manuscript for *Chemistry A European Journal*.: (2*R**,6*R**,1'*S**)-4,4-Dimethyl-6-phenyl-2-(2,2,2-trichloro-1-hydroxy-ethyl)-1,3,2-dioxaphosphinane- 2-oxide (**24c**).

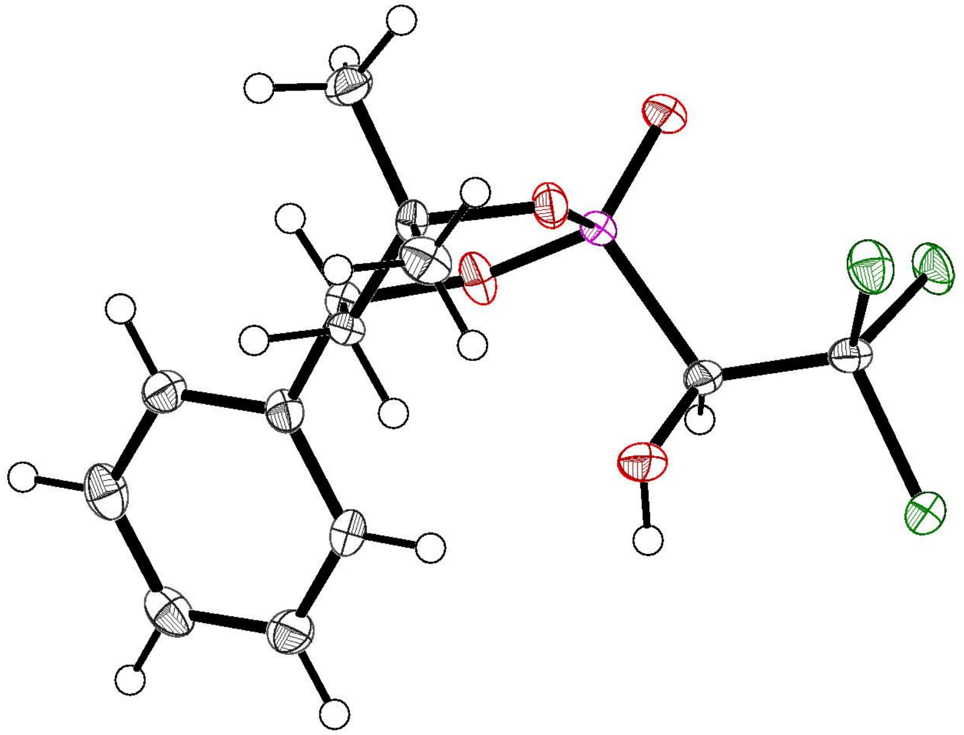

Figure 3. Molecular structure of (**24c**), drawn with 50% displacement ellipsoids. Two different hydrogen bond distances are recorded. The donor-acceptor distances are 2.6462(13) Å and 2.8130(13) Å. They spread mostly along axis b. Taking into account the corresponding angles of 161.9 (8) ° and 163.9 (9) °and following the classification of Jeffrey[6] it can be supposed that these two intermolecular hydrogen bonds are of moderate character. Several parameter values indicate small quality leaks in the measurement interpretation! Especially the second weighting scheme parameter with 27.8746 and the GOF with 1.292 are higher than for a "good" result expected. Some rest electron density peaks, values close to 0.7 eÅ^{-3}, form a small percentage of possible disorder in the structure! We were not able to interpret this possible disorder reliable. Due to the quality of the NMR result, the synthetic path and the very high ratio of the main part, interpretable in only one way, there is no need for a new measurement.

**Chemical formula**	C13H16Cl3O4P	**Crystal system**	monoclinic	
**Formula weight**	373.58	**Space group**	*C 1 2/c 1*	
**Temperature**	100(2) K	**Z**	8	
**Measurement method**	\Φ and \ω scans	**Volume**	3192.2(3) Å^3	
**Radiation**	Mo K\α	**Unit cell dimensions**	a = 19.0063(9) Å	α = 90°
**Wavelength**	0.71073 Å			
**Crystal size**	0.183 x 0.075 x 0.045 mm^3		b = 6.0646(3) Å	β = 93.4956(14)°
**Crystal habit**	clear colorless block		c = 27.7463(15) Å	γ = 90°
**Density (calculated)**	1.555 g/cm^3	**Absorption coefficient**	0.685 mm^{-1}	
**Abs. correction Tmin**	0.6876	**Abs. correction Tmax**	0.7460	
**Abs. correction type**	multi-scan	**F(000)**	1536.0 e$^-$	

Table 6. Sample and crystal data of (**24c**).

**Theta range for data collection**	1.471to 25.344°		**Index ranges**	-22<=h<=22, -7<=k<=7 , -33<=l<=33
**Refinement method**	Least squares		**Function minimized**	$\Sigma\ w(F_o^2 - F_c^2)^2$
**Reflections number**	31746			
**Data / restraints / parameters**	2914 / 0 / 183		**Goodness-of-fit on F^2**	1.292
**Final R indices**	2819 data; I>2σ(I)	R1 = 0.0591, wR2 = 0.1417	all data (2914)	R1 = 0.0606, wR2 = 0.1423
**Weighting scheme**	$w=1/[\sigma^2(F_o^2)+(0.0364P)^2+27.8746P]$		**Largest diff. peak and hole**	0.783 and -0.403eÅ^{-3}
	where $P=(F_o^2+2F_c^2)/3$		**R.M.S. deviation from mean**	0.096 eÅ^{-3}

Table 7. Data collection and structure refinement of (**24c**).

4) Compound (25a) submitted for X-ray structure analysis

Compound name and number in manuscript for *Chemistry A European Journal*.: (2*S**,6*R**)-2-(2,2-Dichlorovinyloxy)-4,4-dimethyl-6-phenyl-1,3,2-dioxaphosphinane-2-oxide (**25a**).

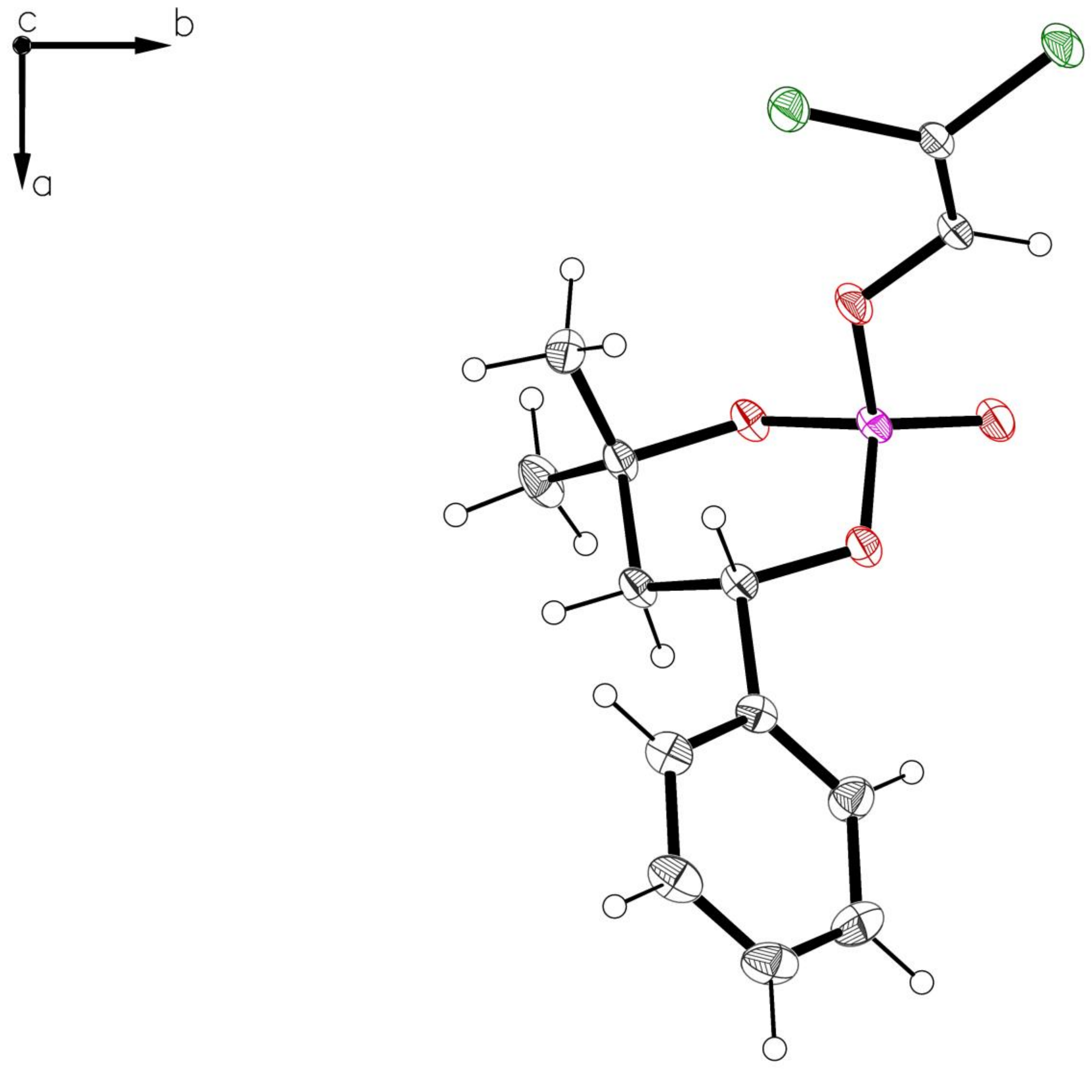

Figure 4. Molecular structure of (**25a**), drawn with 50% displacement ellipsoids. The highlighted molecule corresponds to the asymmetric unit. One hydrogen bond distance is recorded. The intermolecular donor-acceptor (N-H··· O) distance is 2.8694(2) Å. Taking into account the corresponding angle of 134.232 (3) ° and following the classification of Jeffrey[6] it can be supposed that this hydrogen bond is of moderate character. It is worth mentioning that in this structure the center of symmetry is located close to the hydrogen bond. Due to this the two illustrated bonds are identical likewise the two visible molecules.

**Chemical formula**	C13H15Cl2O4P	**Crystal system**	monoclinic	
**Formula weight**	337.12	**Space group**	*P 1 2_1/c 1*	
**Temperature**	100(2) K	**Z**	4	
**Measurement method**	\Φ and \ω scans	**Volume**	1523.38(11) $Å^3$	
**Radiation**	Mo K\α	**Unit cell dimensions**	a = 12.3049(5) Å	α = 90°
**Wavelength**	0.71073 Å			
**Crystal size**	0.146 x 0.117 x 0.052 mm^3		b = 12.5109(5) Å	β = 101.6632(12)°
**Crystal habit**	clear colorless block		c = 10.1042(4) Å	γ = 90°
**Density (calculated)**	1.470 g/cm^3	**Absorption coefficient**	0.540 mm^{-1}	
**Abs. correction Tmin**	0.7163	**Abs. correction Tmax**	0.7460	
**Abs. correction type**	multi-scan	**F(000)**	696.0 e^-	

Table 8. Sample and crystal data of (**25a**).

**Theta range for data collection**	1.690 to 30.034°		**Index ranges**	-17<=h<=17,-17<=k<=17, -14<=l<=14
**Refinement method**	Least squares		**Function minimized**	Σ w(F_o^2 - F_c^2)^2
**Reflections number**	40983			
**Data / restraints / parameters**	4462 / 0 / 183		**Goodness-of-fit on F^2**	1.041
**Final R indices**	3969 data; I>2σ(I)	R1 = 0.0262, wR2 = 0.0687	all data (4462)	R1 = 0.0312, wR2 = 0.0716
**Weighting scheme**	w=1/[σ^2(F_o^2)+(0.0364P)^2+0.6288P]		**Largest diff. peak and hole**	0.467 and -0.278e$Å^{-3}$
	where P=(F_o^2+2F_c^2)/3		**R.M.S. deviation from mean**	0.054 e$Å^{-3}$

Table 9. Data collection and structure refinement of (**25a**).

References

[1] Bruker SAINT V7.68A Copyright © 2005-2013 Bruker AXS

[2] G. M. Sheldrick **1996**. *Program name.* University of Göttingen, Germany.

[3] G.M. Sheldrick *Acta Cryst. A64* **2008**, 112-122.

[4] O.V. Dolomanov, L. J. Bourhis, R. J. Gildea, J. A. K. Howard, H. Puschmann, *J. Appl. Cryst.* **2009,** *42*, 339-341.

[5] C. B. Huebschle, G. M. Sheldrick, B. Dittrich, *J. Appl. Cryst*. **2011**, *44*, 1281-1284.
